# The Impact of Non-coding RNAs in the Epithelial to Mesenchymal Transition

**DOI:** 10.3389/fmolb.2021.665199

**Published:** 2021-03-26

**Authors:** Bashdar Mahmud Hussen, Hamed Shoorei, Mahdi Mohaqiq, Marcel E. Dinger, Hazha Jamal Hidayat, Mohammad Taheri, Soudeh Ghafouri-Fard

**Affiliations:** ^1^Pharmacognosy Department, College of Pharmacy, Hawler Medical University, Erbil, Iraq; ^2^Department of Anatomical Sciences, Faculty of Medicine, Birjand University of Medical Sciences, Birjand, Iran; ^3^Wake Forest Institute for Regenerative Medicine, School of Medicine, Wake Forest University, Winston-Salem, NC, United States; ^4^School of Biotechnology and Biomolecular Sciences, University of New South Wales, Sydney, NSW, Australia; ^5^Department of Biology, College of Education, Salahaddin University-Erbil, Erbil, Iraq; ^6^Urology and Nephrology Research Center, Shahid Beheshti University of Medical Sciences, Tehran, Iran; ^7^Department of Medical Genetics, Shahid Beheshti University of Medical Sciences, Tehran, Iran

**Keywords:** lncRNA, miRNA, epithelial to mesenchymal transition, expression, biomarker

## Abstract

Epithelial to mesenchymal transition (EMT) is a course of action that enables a polarized epithelial cell to undertake numerous biochemical alterations that allow it to adopt features of mesenchymal cells such as high migratory ability, invasive properties, resistance to apoptosis, and importantly higher-order formation of extracellular matrix elements. EMT has important roles in implantation and gastrulation of the embryo, inflammatory reactions and fibrosis, and transformation of cancer cells, their invasiveness and metastatic ability. Regarding the importance of EMT in the invasive progression of cancer, this process has been well studies in in this context. Non-coding RNAs (ncRNAs) have been shown to exert critical function in the regulation of cellular processes that are involved in the EMT. These processes include regulation of some transcription factors namely SNAI1 and SNAI2, ZEB1 and ZEB2, Twist, and E12/E47, modulation of chromatin configuration, alternative splicing, and protein stability and subcellular location of proteins. In the present paper, we describe the influence of ncRNAs including microRNAs and long non-coding RNAs in the EMT process and their application as biomarkers for this process and cancer progression and their potential as therapeutic targets.

## Introduction

Epithelial to mesenchymal transition (EMT) is a course of action that permits polarized epithelial cells, that typically interrelate with basement membrane through their basal facet, to undertake numerous biochemical alterations that allow them to adopt features of mesenchymal cells such as high migratory ability, invasive properties, resistance to apoptosis, and importantly the higher-order formation of extracellular matrix elements (Kalluri and Neilson, 2003). The EMT process is completed by the destruction of the basement membranes and development of mesenchymal cells that are able to roam from their original epithelial layer (Roche, 2018). Induction and establishment of the EMT program is associated with activation of several transcription factors and cell-surface markers, reformation and activation of cytoskeletal proteins, synthesis of ECM-degenerating enzymes, and alteration in the expressions of several non-coding RNAs (ncRNAs) (Kalluri and Neilson, 2003; Roche, 2018). At least three types of EMT are recognized. These distinct types are involved in the processes of implantation and gastrulation of embryos, inflammatory responses and fibrosis, and transformation of cancer cells, their invasiveness and metastatic ability, respectively (Kalluri and Neilson, 2003).

## EMT in Physiological Processes

Epithelial to mesenchymal transition has critical roles in generation of various tissues in the course of development of organisms. Importantly, EMT has an indispensable role in the gastrulation of metazoans and delamination of neural crest cells in vertebrate embryos (Thiery et al., 2009). EMT also partakes in wound healing (Kim et al., 2014). In addition, EMT regulates function of embryonic stem cells through various routes (Kim et al., 2014). Conversion of epithelial cells to mesenchymal cells has been detected in the course of differentiation of embryonic stem cells. In humans, differentiation of these cells is achieved through up-regulation of N-cadherin instead of E-cadherin, enhancement of vimentin levels, over-expression of E-cadherin-suppressing molecules including Snail and Slug, and activation of gelatinase and upsurge in motility of cells (Kim et al., 2014).

## EMT in Cancer

In the context of cancer, EMT is activated by several factors such as hypoxia, cytokines, and growth factors. These molecules are produced by numerous cells that are present in the tumor milieu in response to metabolic alteration, innate and adaptive immune reactions, and administration of antitumor drugs (Roche, 2018). EMT is associated with comprehensive changes in the expression profile of genes. This expression switch is accomplished through an integrative regulatory network that consists of a number of transcription factors namely SNAI1 and SNAI2, ZEB1 and ZEB2, Twist, and E12/E47, ncRNAs, and other factors that modulate chromatin configuration, alternative splicing, and protein stability and subcellular location (De Craene and Berx, 2013). The most important feature of EMT is the over-expression of N-cadherin and the subsequent downregulation of E-cadherin (Loh et al., 2019). This process has important implications in the design of anticancer therapeutic agents (Marcucci et al., 2016) and, moreover, has fundamental roles in the metastatic potential of cancer cells, a process whose reversion is critical in cancer treatment (Roche, 2018). Thus, identification of the molecular pathways that control EMT process is a prerequisite for development of novel anticancer therapies. In the current paper, we describe the role of ncRNAs including microRNAs (miRNAs) and long non-coding RNAs (lncRNAs) in the EMT process and their application as biomarkers for this process and cancer progression and their potential as therapeutic targets.

## miRNAs and EMT

miRNAs are transcripts with sizes around 22 to 24 nucleotides. They are principally bind with the 3′ UTR of selected transcripts to suppress their translation or degrade them via slicer-dependent route (Macfarlane and Murphy, 2010). Several miRNAs influence the EMT process in different cancer types. In lung cancer, miR-451a has a central role in blocking EMT and conferring sensitivity to doxorubicin through this mechanism. miR-451a decreases expressions of N-cadherin and Vimentin, whereas it surges expression of E-cadherin. Functional studies show that the direct interaction between miR-451a and c-Myc contributes in blocking EMT and chemoresistance in lung cancer cells (Tao et al., 2020). The well-known oncogenic miRNA miR-21 has a noticeable role in induction of EMT through modulation of the PTEN/Akt/GSK3 beta pathway and regulation of transcription of E-cadherin, vimentin, snail, slug and β-catenin (Dai L. et al., 2019). In prostate cancer patients, expression of miR-210-3p is increased in bone metastatic specimens compared with non-bone metastatic specimens. Up-regulation of this miRNA is associated with PSA concentrations in serum, Gleason grade and metastatic probability to bone in these patients. *In vitro* experiments show the effect of miR-210-3p in augmentation of EMT, invasion and migration of prostate cancer cells. Notably, animal studies show that miR-210-3p knockdown decreases bone metastasis of PC-3 cells. This miRNA preserves the constant induction of NF-κB signaling through modulating expression of SOCS1 and TNIP1 (Ren et al., 2017). Expression of miR-23a is augmented in metastatic breast cancer cells and in patients with lymph node involvement. Notably, expression of this miRNA is increased after treatment of breast cancer cells with TGF-β1. Importantly, both cell line assays and *in vivo* tests show that miR-23a silencing suppressed TGF-β1-stimulated EMT, migration, invasiveness and metastatic probability. The role of miR-23a in EMT is exerted via its binding with CDH1, a critical gene in EMT process. Remarkably, Wnt/β-catenin signaling is also engaged in miR-23a facilitated progression of EMT (Ma et al., 2017). In colorectal cancer, expression of miR-330 has been down-regulated parallel with up-regulation of HMGA2 levels and poor clinical outcome. Stable up-regulation of miR-330 in these cell lines has decreased HMGA2 levels, enhanced apoptosis and decreased migratory potential and viability of these cells. Notably, this miRNA has also reduced expressions of EMT markers including Snail-1, E-cadherin and VEGF as well as some other oncogenic proteins namely SMAD3 and AKT (Mansoori et al., 2020). In colorectal cancer, miR-145-5p, miR-3622a-3p, miR-205 and miR-200b inhibit EMT through targeting CDCA3, SALL4, MDM4 and HIF-1α, respectively (Shang et al., 2017; Chang et al., 2020; Chen et al., 2020; Fan and Wang, 2020).

**FIGURE 1:**
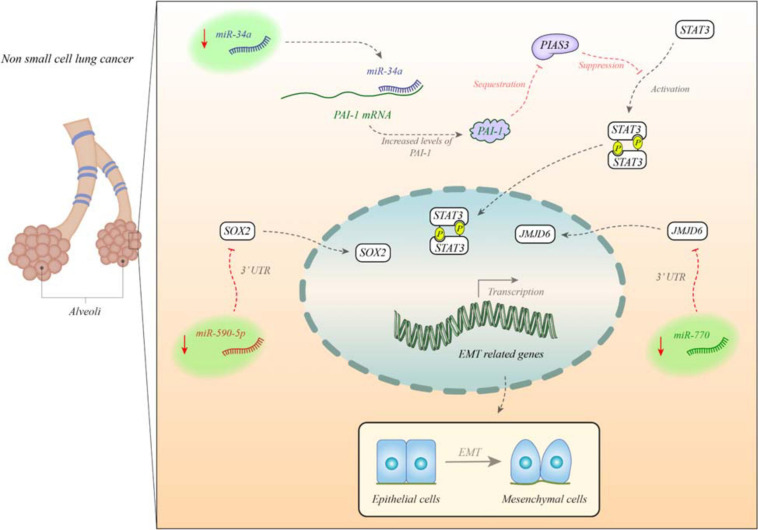
miR-34a is decreased in non-small cell lung cancer (NSCLC). miR-34a blocks expression of PAI. PAI has a role in suppression of PIAS3 expression and blocking its effects on STAT3. STAT3 enhances expression of EMT-related genes (Wang D.X. et al., 2017). In addition, miR-770 and miR-590-5p are decreased in these patients. These miRNA suppress expression of JMJD6 and SOX2, respectively. Under-expression of these miRNAs leads to over-expression of EMT genes.

Figure 1 depicts the impacts of miRNAs in the EMT process in non-small cell lung cancer (NSCLC).

Supplementary Table 1 displays the role of individual miRNAs in the EMT process in diverse human cancers. As EMT has a central part in the progression of cancer, EMT-associated miRNAs have prominent roles in the determination of patients’ survival. For instance, over-expression of miR-200c-3p, miR-99a and miR-92b is linked with prolonged survival in lung cancer, ovarian cancer and breast cancer patients (Li Y.Y. et al., 2019; Zhang L. et al., 2019; Wang H.Y. et al., 2020). Conversely, up-regulation of miR-199b-5p and miR-210-3p is linked with poor survival in prostate cancer patients (Ren et al., 2017; Zhao et al., 2019). Table 1 shows the result of studies that have appraised the prognostic role of EMT-associated miRNAs in diverse cancers.

**TABLE 1:** Prognostic roles of EMT-associated miRNAs in cancer (ACT: adjacent control tissue).

**Sample number**	**Kaplan-Meier analysis**	**References**
70 pairs of LC and ACTs	High miR-200c-3p expression was linked with longer survival.	Wang H.Y. et al., 2020
179 pairs of LC and ACTs	High expression of miR-616-5p was linked with poor overall survival.	Shi et al., 2017
49 pairs of LC and ACTs	Decreased miR-874 expression was linked with poor prognosis.	Wang S. et al., 2020
47 pairs of OC and ACTs	Decreased miR-99a expression in was linked with poor prognosis.	Zhang L. et al., 2019
51 pairs of BC and ACTs	Decreased miR-92b expression was linked with poor prognosis.	Li Y.Y. et al., 2019
60 pairs of BC and ACTs	Decreased miR−516a−3p expression was linked with poor prognosis.	Chi et al., 2019
117 pairs of BLC and ACTs	Decreased miR-221expression was linked with poor prognosis.	Li F. et al., 2019
300 pairs of CRC and ACTs	Decreased miR−330 expression was linked with poor prognosis.	Mansoori et al., 2020
80 pairs of CRC and ACTs	High expression of miR-3622a-3p was linked with better overall survival.	Chang et al., 2020
4 pairs of CRC and ACTs	Decreased miR−598 expression was linked with poor prognosis.	Wang Y. et al., 2017
93 pairs of BC and ACTs	Higher expression of miR-365-3p was linked with better overall survival.	Gao and Tian, 2020
Breast cancer	Higher expression of miR-335 was linked with poor overall survival.	Chen et al., 2019
157 pairs of PaC and ACTs	Decreased miR-3656 expression was linked with poor prognosis.	Yang R.M. et al., 2017
36 OC tissues and 14 normal ovarian tissue	Decreased miR-195-5p expression was linked with poor prognosis.	Dong S. et al., 2019
35 pairs of GC and ACTs	Decreased miR-125a-5p expression was linked with poor prognosis.	Wang X. et al., 2019
52 pairs of CC and ACTs	Decreased miR-31-3p expression was linked with poor prognosis.	Jing et al., 2019
20 pairs of PCa and ACTs	Decreased miR-33a-5p expression was linked with poor prognosis.	Dai Y. et al., 2019
30 pairs of PCa and ACTs	High expression of miR-199b-5p was linked with poor prognosis.	Zhao et al., 2019
52 pairs of PCa and ACTs	High expression of miR-210-3p was linked with poor prognosis.	Ren et al., 2017
60 pairs of OC and ACTs	High expression of miR-1228 was linked with poor prognosis.	Du L. et al., 2020
36 pairs of tumor specimens and adjacent normal specimens	High expression of miR-127 was linked with poor prognosis.	Shi et al., 2017
20 pairs of RCC and ACTs	High expression of miR-452-5p was linked with poor prognosis.	Zhai et al., 2018
36 pairs of GBC and ACTs	Decreased miR-143-5p expression was linked with poor prognosis.	Taheri et al., 2017

## miRNA Roles in EMT in Non-Cancerous Conditions

Expression of miR-29b has been decreased by silica and has affected the mesenchymal-epithelial transition (MET) in RLE-6TN cells. Besides, up-regulation of miR-29b can suppress silica-induced EMT in animals, precluding lung fibrosis, and enhancing respiratory function. Therefore, miR-29b has been suggested as a negative modulator of silicosis fibrosis, possibly through enhancing MET and inhibiting EMT in the lung (Sun et al., 2019). Moreover, miR-200b/c-3p have been shown to modulate epithelial plasticity and suppress skin wound healing through affecting TGF-β-mediated RAC1 signaling (Tang et al., 2020).

## lncRNAs and EMT

LncRNAs are regulatory transcripts with diverse sizes ranging from 200 nucleotides to more than thousands nucleotides. These transcripts regulate expression of genes through altering chromatin configuration, acting as enhances, sponging diverse molecules particularly miRNAs and altering stability of transcripts (Fang and Fullwood, 2016). Through modulation of activity of several cancer-related signaling cascades, lncRNAs modulate metastatic potential of tumor cells (Ghafouri-Fard et al., 2021a,b). Several lncRNAs play a part in the modulation of EMT processes. For instance, expression of NEAT1 is augmented in cervical cancer tissues in correlation with poor survival of patients. This lncRNA directly inhibits expression of miR-361, a miRNA that suppresses HSP90 to impede the invasion and EMT phenotype. Thus, NEAT1 is regarded as a pro-EMT lncRNA in cervical cancer (Xu D. et al., 2020). MALAT1 enhances the EMT features and cisplatin resistance of oral squamous cell carcinoma cells through regulation of the PI3K/AKT/mTOR signaling (Wang R. et al., 2020). In lung and esophageal cancers, MALAT1 exerts similar functions through modulating miR-124 expression and Ezh2/Notch1 axis, respectively (Chen et al., 2018; Wu et al., 2018). On the other hand, MEG3 enhances level of epithelial marker E-cadherin and suppresses mesenchymal markers vimentin and fibronectin in gastric carcinoma cells, indicating an anti-EMT function for this lncRNA (Jiao and Zhang, 2019). In ovarian cancer, TC0101441, CCAT1 and PTAR promote EMT through modulation of KiSS1, miR-490-3p and miR-101-3p, respectively (Liang et al., 2018; Mu et al., 2018; Qiu et al., 2020). Supplementary Table 2 summarizes the functions of EMT-associated lncRNAs in human cancers.

Epithelial to mesenchymal transition-associated lncRNAs have both diagnostic and prognostic values in cancer patients. For example, expression levels of GHET1 could differentiate cancer and normal esophageal tissues with high accuracy (Liu H. et al., 2017). Over-expression of FLVCR1-AS1 and LINC00261 has been associated with poor overall survival rate of patients with neoplasm (Yan et al., 2019; Gao et al., 2020). Table 2 summarizes the results of studies that report diagnostic and prognostic roles of EMT-associated lncRNAs in cancer.

**TABLE 2:** Diagnostic and prognostic role of EMT-associated lncRNAs in cancer (ACTs: adjacent control tissues, OS: overall survival).

**Sample number**	**Area under curve**	**Sensitivity**	**Specificity**	**Kaplan-Meier analysis**	**Multivariate cox regression**	**References**
50 pairs of SOC and ACTs	-	-	-	High expression of FLVCR1-AS1 was linked with poor OS.	High expression of FLVCR1-AS1 was associated with lymphatic metastasis and distant metastasis.	Yan et al., 2019
50 pairs of CCA and ACTs	-	-	-	High expression of LINC00261 was linked with poor OS.	High expression of LINC00261 was associated with large tumor size, positive lymph node metastasis, advanced TNM stages, and higher post-operative recurrence.	Gao et al., 2020
76 pairs of GC and ACTs	-	-	-	High expression of TP73-AS1 was linked with poor OS.	High expression of TP73-AS1 was associated with depth of invasion and TNM stages.	Zhang et al., 2018c
18 pairs of GC and ACTs	-	-	-	Low expression of HRCEG was linked with poor OS.	-	Wu Q. et al., 2020
162 pairs of GC and ACTs	-	-	-	High expression of SNHG7 was linked with poor OS.	High expression of SNHG7 was associated with TNM stage, depth of invasion, lymph-node metastasis, and distant metastasis.	Wu S. et al., 2020
84 pairs of GC and ACTs	-	-	-	High expression of HCP5 was linked with poor OS.	High expression of HCP5 was associated with the size of the tumor, lymph nodes metastasize, and the severity of the disease	Zhang et al., 2020
78 pairs of GC and ACTs	-	-	-	High expression of SNHG6 was linked with poor OS.	High expression of SNHG6 was associated with invasion depth, lymph node metastasis, distant metastasis, and TNM stage.	Yan et al., 2017
92 pairs of CRC and ACTs	-	-	-	High expression of HIF1A-AS2 was linked with poor OS.	High expression of HIF1A-AS2 was associated with TNM stages.	Lin et al., 2018
338 pairs of CRC and ACTs	-	-	-	High expression of SNHG1 was linked with poor OS.	-	Bai et al., 2020
124 pairs of CRC and ACTs	-	-	-	High expression of PANDAR was linked with poor OS.	High expression of PANDAR was associated with tumor diameter, histological differentiation, TNM stage, lymph node metastasis, depth of invasion.	Lu et al., 2017
82 pairs of BC and ACTs	-	-	-	High expression of TP73-AS1 was linked with poor OS.	-	Ding et al., 2019
TCGA database	-	-	-	High expression of PVT1 was linked with poor OS.	-	Chang et al., 2018
40 pairs of HC and ACTs	-	-	-	High expression of SNHG7 was linked with poor OS.	-	Yao et al., 2019
134 pairs of HCC and ACTs	-	-	-	High expression of SBF2-AS1 was linked with poor OS.	High expression of SBF2-AS1was associated with vein invasion and TNM stage.	Zhang et al., 2018e
54 pairs of HCC and ACTs	-	-	-	High expression of LOC105372579 was linked with poor OS.	High expression of LOC105372579 was associated with tumor size and TNM stage.	Changyong et al., 2019
HCC tissues (*n* = 38), normal liver tissues (*n* = 21)	-	-	-	High expression of HULC was linked with poor OS.	High expression of HULC was associated with clinical stage and intrahepatic metastases.	Li et al., 2016
76 pairs of HCC and ACTs	-	-	-	High expression of HOXA−AS3 was linked with poor OS.	-	Tong et al., 2019
76 pairs of OSCC and ACTs	-	-	-	High expression of ADAMTS9-AS2 was linked with poor OS.	High expression of ADAMTS9-AS2 was associated with tumor size, clinical stage, and lymph node metastasis.	Li Y. et al., 2019
123 OSCC tissues and 50 adjacent non-tumor tissues	-	-	-	High expression of H19 was linked with poor OS.	-	Zhang et al., 2017a
128 pairs of BLC and ACTs	-	-	-	High expression of TP73-AS1 was linked with poor OS and PSF rates.	-	Tuo et al., 2018
48 pairs of NPC and ACTs	-	-	-	High expression of TUG1 was linked with poor OS.	-	Qian et al., 2019
42 pairs of BLC and ACTs	-	-	-	High expression of NRON was linked with poor OS.	High expression of NRON was associated with tumor invasion depth.	Xiong et al., 2020
30 pairs of OS and ACTs	-	-	-	High expression of PCAT1 was linked with poor OS.	High expression of PCAT1 was associated with advanced clinical-stage and tumor metastasis.	Zhang et al., 2018d
305 pairs of LUAD and ACTs	-	-	-	High expression of H19 was linked with poor OS.	High expression of H19 was associated with tumor diameter and TNM stage.	Liu et al., 2019
107 pairs of LUAD and ACTs	-	-	-	High expression of TTN-AS1 was linked with poor OS.	High expression of TTN-AS1 was associated with TNM stage and lymph node involvement.	Jia et al., 2019
50 pairs of NSCLC and ACTs	-	-	-	Low expression of NBR2 was linked with poor OS rate.	-	Gao et al., 2019
86 pairs of NSCLC and ACTs	-	-	-	High expression of FEZF1-AS1 was linked with poor OS.	High expression of FEZF1-AS1 was associated with lymph node metastasis, poor differentiation grade, and advanced TNM stage.	He et al., 2017
55 pairs of ESCC and ACTs	0.858	69.7%	91.3%	Low expression of GHET1 was linked with poor OS.	High expression of GHET1 was associated with lymph node metastasis, differentiation, and TNM stage.	Liu H. et al., 2017
25 pairs of RCC and ACTs	-	-	-	High expression of PVT1 was linked with poor OS.	High expression of PVT1 was associated with TNM stage, fuhrman grade, lymph node involvement, and tumor dimension.	Ren et al., 2019

## Discussion

Numerous miRNAs and lncRNAs have been shown to regulate EMT process. These ncRNAs participate in this process through influencing activity of several signaling pathways such as NF-κB, TGF-β, Wnt/β-catenin, Akt/mTOR, PIK3R3 and EGFR. The Wnt/β-catenin pathway is the target of several miRNAs such as miR-6838-5p, miR-770, miR-23a, miR-27a, miR-125b, miR-375, miR−516a−3p, miR-630, miR-330-3p, miR-147, miR-138 and miR-3622a-3p. Moreover, lncRNAs UCA1, SNHG7, GATA6-AS1, CRNDE and FEZF1-AS1 exert their regulatory roles on EMT through modulation of this signaling pathway. Thus, the Wnt/β-catenin pathway can be regarded as a focal point for organization of EMT-associated ncRNAs. This important position potentiates this pathway as a therapeutic target in reversing the EMT process. As the Wnt/β-catenin pathway has been implicated in the progression of EMT during tumor evolution (Basu et al., 2018), it is predicted that ncRNAs contribute to the fine-tuning of activity of this pathway to confer different degrees of EMT.

Circular RNAs are another group of ncRNAs that participate in carcinogenesis (Su et al., 2019). However, their role in the EMT process has been less studied. High−throughput transcript sequencing as a new method can be applied to identify EMT-associated circRNAs. This strategy has led to identification of 7 up-regulated circRNAs and 16 down-regulated circRNAs in breast cancer cells with EMT phenotype. CircSCYL2 has been among under-expressed circRNAs in breast cancer tissues and cell lines. Up-regulation of circSCYL2 has suppressed migration and invasion (Yuan et al., 2020).

Several therapeutic modalities such as short hairpin RNAs and engineered antibodies have been designed to reverse the EMT process in cancer cells. Moreover, a number of natural agents have been demonstrated to suppress EMT through modulation of the important EMT-associated molecules or pathways (Loh et al., 2019). NcRNAs have been involved in the therapeutic efficiency of both conventional and natural anticancer drugs (Dong Y. et al., 2019; Tao et al., 2020). Thus, modulation of expression of EMT-associated ncRNAs is a promising strategy for enhancement of the response of patients to anti-cancer drugs.

Expression levels of EMT-associated miRNAs and lncRNAs has been linked to the survival of cancer patients. Therefore, it is possible that a panel of EMT-associated miRNAs and lncRNAs predict disease progression and therapeutic response with clinically relevant accuracy. However, there is no consensus set of ncRNAs to facilitate the design of such diagnostic tools as yet. Thus, future studies should focus on the integration of data provided by single studies to propose a diagnostic/prognostic panel consisting of EMT-associated lncRNAs and miRNAs. As discussed above, lncRNAs and miRNAs have functional interactions to modulate EMT. System biology methods are useful in recognition of such interactions and depicting the interaction network to identify the most important modules. Identification of these modules not only facilitates design of diagnostic panels, but also help in design of targeted therapies. Systems biology methods have been successfully used to integrate modeling and experimental data, leading to identification of several intermediate states participating in the EMT process (Hong et al., 2015). Moreover, construction of model of the miRNA-based coupled chimeric modules has led to identification of the role of miR-200/ZEB module in switching between epithelial and mesenchymal features and in establishment of a hybrid phenotype with assorted features of collective cell migration, as documented in physiological processes (Lu et al., 2013). Moreover, system biology methods have been used to find the main regulatory network which controls TGF-β-induced EMT (Tian et al., 2013).

Taken together, ncRNAs are associated with important features in invasive and metastatic cancers, i.e., the EMT process. Therapeutic interventions that modulate expression of these transcripts can improve survival of cancer patients.

Although the role of ncRNAs in regulation of EMT in cancer has been extensively appraised, less is known about their contribution in the regulation of this process in non-cancerous context.

## Author Contributions

MT and SG-F wrote the draft and revised it. HS, MM, MD, and HH collected the data, designed the tables and figures. All the authors approved the submitted version.

## Conflict of Interest

The authors declare that the research was conducted in the absence of any commercial or financial relationships that could be construed as a potential conflict of interest.

## References

[B1] Amaar Y. G. Reeves M. E. (2019). RASSF1C regulates miR-33a and EMT marker gene expression in lung cancer cells. *Oncotarget* 10 123–132. 10.18632/oncotarget.26498 30719208 PMC6349430

[B2] Bai J. Xu J. Zhao J. Zhang R. (2020). lncRNA SNHG1 cooperated with miR−497/miR−195−5p to modify epithelial–mesenchymal transition underlying colorectal cancer exacerbation. *J. Cell. Physiol.* 235 1453–1468. 10.1002/jcp.29065 31276207

[B3] Bai Z. Wang J. Wang T. Li Y. Zhao X. Wu G. (2017). The MiR-495/Annexin A3/P53 axis inhibits the invasion and EMT of colorectal cancer cells. *Cell Physiol. Biochem.* 44 1882–1895. 10.1159/000485877 29224019

[B4] Basu S. Chaudhary A. Chowdhury P. Karmakar D. Basu K. Karmakar D. (2020). Evaluating the role of hsa-miR-200c in reversing the epithelial to mesenchymal transition in prostate cancer. *Gene* 730:144264. 10.1016/j.gene.2019.144264 31759982

[B5] Basu S. Cheriyamundath S. Ben-Ze’ev A. (2018). Cell-cell adhesion: linking Wnt/β-catenin signaling with partial EMT and stemness traits in tumorigenesis. *F1000Res.* 7:F1000FacultyRev–488. 10.12688/f1000research.15782.1 PMC6144947 30271576

[B6] Cao W. Zhou G. (2020). LncRNA SNHG12 contributes proliferation, invasion and epithelial-mesenchymal transition of pancreatic cancer cells by absorbing miRNA-320b. *Biosci. Rep.* 40:BSR20200805. 10.1042/BSR20200805 PMC7276652 32432698

[B7] Cao Y. Shen T. Zhang C. Zhang Q. H. Zhang Z. Q. (2019). MiR-125a-5p inhibits EMT of ovarian cancer cells by regulating TAZ/EGFR signaling pathway. *Eur. Rev. Med. Pharmacol. Sci.* 23 8249–8256. 31646555 10.26355/eurrev_201910_19134

[B8] Chang S. Sun G. Zhang D. Li Q. Qian H. (2020). MiR-3622a-3p acts as a tumor suppressor in colorectal cancer by reducing stemness features and EMT through targeting spalt-like transcription factor 4. *Cell Death Dis.* 11:592 10.1038/s41419-020-02789-z PMC7385142 32719361

[B9] Chang Z. (2019). Downregulation of SOX2 may be targeted by miR-590-5p and inhibits epithelial-to-mesenchymal transition in non-small-cell lung cancer. *Exp. Ther. Med.* 18 1189–1195. 31316613 10.3892/etm.2019.7642 PMC6601398

[B10] Chang Z. Cui J. Song Y. (2018). Long noncoding RNA PVT1 promotes EMT via mediating microRNA-186 targeting of Twist1 in prostate cancer. *Gene* 654 36–42. 10.1016/j.gene.2018.02.036 29452232

[B11] Changyong E. Yang J. Li H. Li C. (2019). LncRNA LOC105372579 promotes proliferation and epithelial-mesenchymal transition in hepatocellular carcinoma via activating miR-4316/FOXP4 signaling. *Cancer Manag Res.* 11 2871–2879. 10.2147/cmar.s197979 31114338 PMC6489618

[B12] Chen J. H. Huang W. C. Bamodu O. A. Chang P. M. Chao T. Y. Huang T. H. (2019). Monospecific antibody targeting of CDH11 inhibits epithelial-to-mesenchymal transition and represses cancer stem cell-like phenotype by up-regulating miR-335 in metastatic breast cancer, in vitro and in vivo. *BMC Cancer* 19:634. 10.1186/s12885-019-5811-1 31248373 PMC6598338

[B13] Chen M. Xia Z. Chen C. Hu W. Yuan Y. (2018). LncRNA MALAT1 promotes epithelial-to-mesenchymal transition of esophageal cancer through Ezh2-Notch1 signaling pathway. *Anticancer Drugs* 29 767–773. 10.1097/cad.0000000000000645 29916899

[B14] Chen Q. Zhou L. Ye X. Tao M. Wu J. (2020). miR-145-5p suppresses proliferation, metastasis and EMT of colorectal cancer by targeting CDCA3. *Pathol. Res. Pract.* 216:152872. 10.1016/j.prp.2020.152872 32107086

[B15] Cheng Y. Chang Q. Zheng B. Xu J. Li H. Wang R. (2019). LncRNA XIST promotes the epithelial to mesenchymal transition of retinoblastoma via sponging miR-101. *Eur. J. Pharmacol.* 843 210–216. 10.1016/j.ejphar.2018.11.028 30472203

[B16] Chi Y. Wang F. Zhang T. Xu H. Zhang Y. Shan Z. (2019). miR-516a-3p inhibits breast cancer cell growth and EMT by blocking the Pygo2/Wnt signalling pathway. *J. Cell. Mol. Med.* 23 6295–6307. 10.1111/jcmm.14515 31273950 PMC6714144

[B18] Dai L. Chen F. Zheng Y. Zhang D. Qian B. Ji H. (2019). miR-21 regulates growth and EMT in lung cancer cells via PTEN/Akt/GSK3beta signaling. *Front. Biosci.* 24:1426–1439. 10.2741/4788 31136988

[B19] Dai Y. Wu Z. Lang C. Zhang X. He S. Yang Q. (2019). Copy number gain of ZEB1 mediates a double-negative feedback loop with miR-33a-5p that regulates EMT and bone metastasis of prostate cancer dependent on TGF-beta signaling. *Theranostics* 9 6063–6079. 10.7150/thno.36735 31534537 PMC6735523

[B20] De Craene B. Berx G. (2013). Regulatory networks defining EMT during cancer initiation and progression. *Nat. Rev. Cancer* 13 97–110. 10.1038/nrc3447 23344542

[B21] Ding J. Wu W. Yang J. Wu M. (2019). Long non-coding rna mif-as1 promotes breast cancer cell proliferation, migration and emt process through regulating mir-1249-3p/hoxb8 axis. *Pathol. Res. Pract.* 215:152376. 10.1016/j.prp.2019.03.005 31097355

[B22] Ding Q. Mo F. Cai X. Zhang W. Wang J. Yang S. (2020). LncRNA CRNDE is activated by SP1 and promotes osteosarcoma proliferation, invasion, and epithelial−mesenchymal transition via Wnt/β−catenin signaling pathway. *J. Cell. Biochem.* 121 3358–3371. 10.1002/jcb.29607 31898343

[B23] Dong S. Wang R. Wang H. Ding Q. Zhou X. Wang J. (2019). HOXD-AS1 promotes the epithelial to mesenchymal transition of ovarian cancer cells by regulating miR-186-5p and PIK3R3. *J. Exp. Clin. Cancer Res.* 38:110. 10.1186/s13046-019-1103-5 PMC6397490 30823895

[B24] Dong Y. Chen H. Gao J. Liu Y. Li J. Wang J. (2019). Bioactive ingredients in chinese herbal medicines that target non-coding RNAs: promising new choices for disease treatment. *Front. Pharmacol.* 10:515. 10.3389/fphar.2019.00515 31178721 PMC6537929

[B25] Dou C. Liu Z. Xu M. Jia Y. Wang Y. Li Q. (2016). miR-187-3p inhibits the metastasis and epithelial-mesenchymal transition of hepatocellular carcinoma by targeting S100A4. *Cancer Lett.* 381 380–390. 10.1016/j.canlet.2016.08.011 27544906

[B26] Du L. Xu Z. Wang X. Liu F. (2020). Integrated bioinformatics analysis identifies microRNA-376a-3p as a new microRNA biomarker in patient with coronary artery disease. *Am. J. Transl. Res.* 12:633. PMC7061823 32194911

[B27] Du W. Sun L. Liu T. Zhu J. Zeng Y. Zhang Y. (2020). The miR6253p/AXL axis induces nonT790M acquired resistance to EGFRTKI via activation of the TGFbeta/Smad pathway and EMT in EGFRmutant nonsmall cell lung cancer. *Oncol Rep.* 44 185–195. 32319651 10.3892/or.2020.7579 PMC7251657

[B29] Fan Y. Wang K. (2020). miR-205 suppresses cell migration, invasion and EMT of colon cancer by targeting mouse double minute 4. *Mol. Med. Rep.* 22 633–642. 10.3892/mmr.2020.11150 32467998 PMC7339668

[B30] Fang Y. Fullwood M. J. (2016). Roles, functions, and mechanisms of long non-coding RNAs in cancer. *Genomics Proteomics Bioinform.* 14 42–54. 10.1016/j.gpb.2015.09.006 26883671 PMC4792843

[B31] Fang Y. Y. Tan M. R. Zhou J. Liang L. Liu X. Y. Zhao K. (2019). miR-214-3p inhibits epithelial-to-mesenchymal transition and metastasis of endometrial cancer cells by targeting TWIST1. *Onco Targets Ther.* 12 9449–9458. 10.2147/ott.s181037 31819476 PMC6875683

[B32] Feng S. Yao J. Zhang Z. Zhang Y. Zhang Z. Liu J. (2018). miR96 inhibits EMT by targeting AEG1 in glioblastoma cancer cells. *Mol. Med. Rep.* 17 2964–2972. 29257267 10.3892/mmr.2017.8227 PMC5783515

[B33] Gao F. Tian J. (2020). FOXK1, regulated by miR-365-3p, promotes cell growth and EMT indicates unfavorable prognosis in breast cancer. *Onco Targets Ther.* 13 623–634. 10.2147/ott.s212702 32021304 PMC6982530

[B34] Gao J. Qin W. Kang P. Xu Y. Leng K. Li Z. (2020). Up-regulated LINC00261 predicts a poor prognosis and promotes a metastasis by EMT process in cholangiocarcinoma. *Pathol. Res. Pract.* 216:152733. 10.1016/j.prp.2019.152733 31812439

[B35] Gao Y. Li Y. Li H. Zhao B. (2019). LncRNA NBR2 inhibits EMT progression by regulating Notch1 pathway in NSCLC. *Eur. Rev. Med. Pharmacol. Sci.* 23 7950–7958. 31599420 10.26355/eurrev_201909_19011

[B36] Ghafouri-Fard S. Abak A. Bahroudi Z. Shoorei H. Abbas Raza S. H. Taheri M. (2021a). The interplay between non-coding RNAs and Twist1 signaling contribute to human disorders. *Biomed Pharmacother.* 135:111220. 10.1016/j.biopha.2021.111220 33433357

[B37] Ghafouri-Fard S. Abak A. Tondro Anamag F. Shoorei H. Majidpoor J. Taheri M. (2021b). The emerging role of non-coding RNAs in the regulation of PI3K/AKT pathway in the carcinogenesis process. *Biomed Pharmacother.* 137:111279. 10.1016/j.biopha.2021.111279 33493969

[B38] Gong J. Wang Y. Shu C. (2020). LncRNA CHRF promotes cell invasion and migration via EMT in gastric cancer. *Eur. Rev. Med. Pharmacol. Sci.* 24 1168–1176. 32096147 10.26355/eurrev_202002_20168

[B39] Guo F. Gao Y. Sui G. Jiao D. Sun L. Fu Q. (2019). miR-375-3p/YWHAZ/beta-catenin axis regulates migration, invasion, EMT in gastric cancer cells. *Clin. Exp. Pharmacol. Physiol.* 46 144–152. 10.1111/1440-1681.13047 30353914

[B40] Guo W. Jiang H. Li H. Li F. Yu Q. Liu Y. (2019). LncRNA-SRA1 suppresses osteosarcoma cell proliferation while promoting cell apoptosis. *Technol Cancer Res. Treat.* 18:1533033819841438. 10.1177/1533033819841438 PMC6535715 31106680

[B41] He R. Zhang F. H. Shen N. (2017). LncRNA FEZF1-AS1 enhances epithelial-mesenchymal transition (EMT) through suppressing E-cadherin and regulating WNT pathway in non-small cell lung cancer (NSCLC). *Biomed Pharmacother.* 95 331–338. 10.1016/j.biopha.2017.08.057 28858731

[B42] Hong T. Watanabe K. Ta C. H. Villarreal-Ponce A. Nie Q. Dai X. (2015). An Ovol2-Zeb1 mutual inhibitory circuit governs bidirectional and multi-step transition between epithelial and mesenchymal states. *PLoS Comput. Biol.* 11:e1004569. 10.1371/journal.pcbi.1004569 26554584 PMC4640575

[B43] Hou Y. (2019). MiR-506 inhibits cell proliferation, invasion, migration and epithelial-to-mesenchymal transition through targeting RWDD4 in human bladder cancer. *Oncol. Lett.* 17 73–78. 30655740 10.3892/ol.2018.9594 PMC6313135

[B44] Hu H. (2019). Knockdown of LncRNA SNHG7 inhibited epithelial-mesenchymal transition in prostate cancer though miR-324-3p/WNT2B axis in vitro. *Pathol.Res. Pract.* 215:152537. 10.1016/j.prp.2019.152537 31324390

[B45] Hu Q. Yin J. Zeng A. Jin X. Zhang Z. Yan W. (2018). H19 functions as a competing endogenous RNA to regulate EMT by sponging miR-130a-3p in Glioma. *Cell Physiol. Biochem.* 50 233–245. 10.1159/000494002 30282068

[B46] Hu W. Yan F. Ru Y. Xia M. Yan G. Zhang M. (2020). MIIP inhibits EMT and cell invasion in prostate cancer through miR-181a/b-5p-KLF17 axis. *Am. J. Cancer Res.* 10 630–647. 32195032 PMC7061746

[B47] Huang J. He Y. McLeod H. L. Xie Y. Xiao D. Hu H. (2017). miR-302b inhibits tumorigenesis by targeting EphA2 via Wnt/beta-catenin/EMT signaling cascade in gastric cancer. *BMC Cancer* 17:886. 10.1186/s12885-017-3875-3 29273006 PMC5741943

[B48] Huang R. Li J. Pan F. Zhang B. Yao Y. (2020). The activation of GPER inhibits cells proliferation, invasion and EMT of triple-negative breast cancer via CD151/miR-199a-3p bio-axis. *Am. J. Transl. Res.* 12 32–44. 32051735 PMC7013229

[B49] Ji L. Li X. Zhou Z. Zheng Z. Jin L. Jiang F. (2020). LINC01413/hnRNP-K/ZEB1 axis accelerates cell proliferation and emt in colorectal cancer via inducing YAP1/TAZ1 translocation. *Mol. Ther. Nucleic Acids* 19:546. 10.1016/j.omtn.2019.11.027 31927328 PMC6953771

[B50] Jia Y. Duan Y. Liu T. Wang X. Lv W. Wang M. (2019). LncRNA TTN-AS1 promotes migration, invasion, and epithelial mesenchymal transition of lung adenocarcinoma via sponging miR-142-5p to regulate CDK5. *Cell Death Dis.* 10:573. 10.1038/s41419-019-1811-y PMC6667499 31363080

[B51] Jiang G. Shi W. Fang H. Zhang X. (2018). miR27a promotes human breast cancer cell migration by inducing EMT in a FBXW7dependent manner. *Mol. Med. Rep.* 18 5417–5426. 30365154 10.3892/mmr.2018.9587 PMC6236270

[B52] Jiang Y. Cao W. Wu K. Qin X. Wang X. Li Y. (2019). LncRNA LINC00460 promotes EMT in head and neck squamous cell carcinoma by facilitating peroxiredoxin-1 into the nucleus. *J. Exp. Clin. Cancer Res.* 38:365. 10.1186/s13046-019-1364-z PMC6700841 31429766

[B53] Jiao D. Chen J. Li Y. Tang X. Wang J. Xu W. (2018). miR-1-3p and miR-206 sensitizes HGF-induced gefitinib-resistant human lung cancer cells through inhibition of c-Met signalling and EMT. *J. Cell. Mol. Med.* 22 3526–3536. 10.1111/jcmm.13629 29664235 PMC6010770

[B54] Jiao J. Zhang S. (2019). Long non-coding RNA MEG-3 suppresses gastric carcinoma cell growth, invasion and migration via EMT regulation. *Mol. Med. Rep.* 20 2685–2693. 31524253 10.3892/mmr.2019.10515 PMC6691256

[B56] Jin T. Zhang Y. Zhang T. (2020). MiR-524-5p suppresses migration, invasion, and EMT progression in breast cancer cells through targeting FSTL1. *Cancer Biother. Radiopharm.* 35 789–801. 10.1089/cbr.2019.3046 32298609

[B57] Jing L. Bo W. Yourong F. Tian W. Shixuan W. Mingfu W. (2019). Sema4C mediates EMT inducing chemotherapeutic resistance of miR-31-3p in cervical cancer cells. *Sci. Rep.* 9 17727. 10.1038/s41598-019-54177-z PMC6881343 31776419

[B58] Kalluri R. Neilson E. G. (2003). Epithelial-mesenchymal transition and its implications for fibrosis. *J. Clin. Invest.* 112 1776–1784. 10.1172/jci200320530 14679171 PMC297008

[B59] Kim Y.-S. Yi B.-R. Kim N.-H. Choi K.-C. (2014). Role of the epithelial–mesenchymal transition and its effects on embryonic stem cells. *Exp. Mol. Med.* 46:e108. 10.1038/emm.2014.44 25081188 PMC4150931

[B60] Kong J. Sun W. Li C. Wan L. Wang S. Wu Y. (2016). Long non-coding RNA LINC01133 inhibits epithelial–mesenchymal transition and metastasis in colorectal cancer by interacting with SRSF6. *Cancer Lett.* 380 476–484. 10.1016/j.canlet.2016.07.015 27443606

[B61] Kumar K. J. S. Vani M. G. Hsieh H. W. Lin C. C. Wang S. Y. (2019). Antcin-A modulates epithelial-to-mesenchymal transition and inhibits migratory and invasive potentials of human breast cancer cells via p53-Mediated miR-200c activation. *Planta Med.* 85 755–765. 10.1055/a-0942-2087 31185503

[B62] Lei H. Gao Y. Xu X. (2017). LncRNA TUG1 influences papillary thyroid cancer cell proliferation, migration and EMT formation through targeting miR-145. *Acta Biochim. Biophys. Sin.* 49 588–597. 10.1093/abbs/gmx047 28645161

[B63] Li F. Wang Y. Yang H. Xu Y. Zhou X. Zhang X. (2019). The effect of BACE1-AS on β-amyloid generation by regulating BACE1 mRNA expression. *BMC Mol. Biol.* 20:23. 10.1186/s12867-019-0140-0 31570097 PMC6771094

[B64] Li J. Zhang B. Cui J. Liang Z. Liu K. (2019). miR-203 inhibits the invasion and EMT of gastric cancer cells by directly targeting annexin A4. *Oncol. Res.* 27 789–799. 10.3727/096504018x15444387696532 30837034 PMC7848421

[B66] Li S.-P. Xu H.-X. Yu Y. He J.-D. Wang Z. Xu Y.-J. (2016). LncRNA HULC enhances epithelial-mesenchymal transition to promote tumorigenesis and metastasis of hepatocellular carcinoma via the miR-200a-3p/ZEB1 signaling pathway. *Oncotarget* 7:42431. 10.18632/oncotarget.9883 27285757 PMC5173146

[B67] Li W. Jia G. Qu Y. Du Q. Liu B. Liu B. (2017). Long non-coding RNA (LncRNA) HOXA11-AS promotes breast cancer invasion and metastasis by regulating epithelial-mesenchymal transition. *Med. Sci. Monit. Int. Med. J. Exp. Clin. Res.* 23:3393. 10.12659/msm.904892 28701685 PMC5521048

[B68] Li X. Hou L. Yin L. Zhao S. (2020b). LncRNA XIST interacts with miR-454 to inhibit cells proliferation, epithelial mesenchymal transition and induces apoptosis in triple-negative breast cancer. *J. Biosci.* 45:45. 32098924

[B70] Li Y. Wan Q. Wang W. Mai L. Sha L. Mashrah M. (2019). LncRNA ADAMTS9-AS2 promotes tongue squamous cell carcinoma proliferation, migration and EMT via the miR-600/EZH2 axis. *Biomed. Pharmacother.* 112:108719. 10.1016/j.biopha.2019.108719 30970517

[B71] Li Y. Y. Zheng X. H. Deng A. P. Wang Y. Liu J. Zhou Q. (2019). MiR-92b inhibited cells EMT by targeting Gabra3 and predicted prognosis of triple negative breast cancer patients. *Eur. Rev. Med. Pharmacol. Sci.* 23 10433–10442. 31841197 10.26355/eurrev_201912_19682

[B72] Li Z. Li Y. Wang Y. (2019). miR-19a promotes invasion and epithelial to mesenchymal transition of bladder cancer cells by targeting RhoB. *J BUON.* 24 797–804. 31128038

[B73] Li Z. Tang Y. Xing W. Dong W. Wang Z. (2018). LncRNA, CRNDE promotes osteosarcoma cell proliferation, invasion and migration by regulating Notch1 signaling and epithelial-mesenchymal transition. *Exp. Mol. Pathol.* 104 19–25. 10.1016/j.yexmp.2017.12.002 29246789

[B75] Liang H. Yu M. Yang R. Zhang L. Zhang L. Zhu D. (2020). A PTAL-miR-101-FN1 axis promotes EMT and invasion-metastasis in serous ovarian cancer. *Mol. Ther. Oncolyt.* 16 53–62. 10.1016/j.omto.2019.12.002 31930166 PMC6951825

[B76] Liang H. Yu T. Han Y. Jiang H. Wang C. You T. (2018). LncRNA PTAR promotes EMT and invasion-metastasis in serous ovarian cancer by competitively binding miR-101-3p to regulate ZEB1 expression. *Mol. Cancer* 17 1–13. 30098599 10.1186/s12943-018-0870-5 PMC6087007

[B77] Lin J. Shi Z. Yu Z. He Z. (2018). LncRNA HIF1A-AS2 positively affects the progression and EMT formation of colorectal cancer through regulating miR-129-5p and DNMT3A. *Biomed. Pharmacother.* 98 433–439. 10.1016/j.biopha.2017.12.058 29278853

[B79] Liu H. Zhen Q. Fan Y. (2017). LncRNA GHET1 promotes esophageal squamous cell carcinoma cells proliferation and invasion via induction of EMT. *Int. J. Biol. Mark.* 32 403–408. 10.5301/ijbm.5000304 28983895

[B80] Liu L. Liu L. Lu S. (2019). lncRNA H19 promotes viability and epithelial-mesenchymal transition of lung adenocarcinoma cells by targeting miR-29b-3p and modifying STAT3. *Int. J. Oncol.* 54 929–941. 30747209 10.3892/ijo.2019.4695 PMC6365046

[B83] Liu W. Zhang B. Xu N. Wang M. J. Liu Q. (2017). miR-326 regulates EMT and metastasis of endometrial cancer through targeting TWIST1. *Eur. Rev. Med. Pharmacol. Sci.* 21 3787–3793. 28975990

[B84] Liu Z. Long J. Du R. Ge C. Guo K. Xu Y. (2016). miR-204 regulates the EMT by targeting snai1 to suppress the invasion and migration of gastric cancer. *Tumour Biol.* 37 8327–8335. 10.1007/s13277-015-4627-0 26729198

[B85] Loh C.-Y. Chai J. Y. Tang T. F. Wong W. F. Sethi G. Shanmugam M. K. (2019). The E-Cadherin and N-Cadherin switch in epithelial-to-mesenchymal transition: signaling, therapeutic implications, and challenges. *Cells* 8:1118. 10.3390/cells8101118 31547193 PMC6830116

[B86] Lu M. Jolly M. K. Levine H. Onuchic J. N. Ben-Jacob E. (2013). MicroRNA-based regulation of epithelial–hybrid–mesenchymal fate determination. *Proc. Natl. Acad. Sci. U.S.A.* 110 18144–18149. 10.1073/pnas.1318192110 24154725 PMC3831488

[B87] Lu M. Liu Z. Li B. Wang G. Li D. Zhu Y. (2017). The high expression of long non-coding RNA PANDAR indicates a poor prognosis for colorectal cancer and promotes metastasis by EMT pathway. *J. Cancer Res. Clin. Oncol.* 143 71–81. 10.1007/s00432-016-2252-y 27629879 PMC11818975

[B88] Luo J. Chen J. Li H. Yang Y. Yun H. Yang S. (2017). LncRNA UCA1 promotes the invasion and EMT of bladder cancer cells by regulating the miR-143/HMGB1 pathway. *Oncol. Lett.* 14 5556–5562. 29113184 10.3892/ol.2017.6886 PMC5662909

[B89] Ma F. Li W. Liu C. Li W. Yu H. Lei B. (2017). MiR-23a promotes TGF-beta1-induced EMT and tumor metastasis in breast cancer cells by directly targeting CDH1 and activating Wnt/beta-catenin signaling. *Oncotarget* 8 69538–69550. 10.18632/oncotarget.18422 29050223 PMC5642498

[B90] Macfarlane L.-A. Murphy P. R. (2010). MicroRNA: biogenesis, function and role in cancer. *Curr. Genomics* 11 537–561. 10.2174/138920210793175895 21532838 PMC3048316

[B91] Mansoori B. Mohammadi A. Naghizadeh S. Gjerstorff M. Shanehbandi D. Shirjang S. (2020). miR-330 suppresses EMT and induces apoptosis by downregulating HMGA2 in human colorectal cancer. *J. Cell Physiol.* 235 920–931. 10.1002/jcp.29007 31241772

[B92] Manvati S. Mangalhara K. C. Kalaiarasan P. Chopra R. Agarwal G. Kumar R. (2019). miR-145 supports cancer cell survival and shows association with DDR genes, methylation pattern, and epithelial to mesenchymal transition. *Cancer Cell Int.* 19:230. 10.1186/s12935-019-0933-8 PMC6731614 31516387

[B93] Marcucci F. Stassi G. De Maria R. (2016). Epithelial–mesenchymal transition: a new target in anticancer drug discovery. *Nat. Rev. Drug Discov.* 15:311. 10.1038/nrd.2015.13 26822829

[B94] Mitobe Y. Ikeda K. Sato W. Kodama Y. Naito M. Gotoh N. (2020). Proliferation−associated long noncoding RNA, TMPO−AS1, is a potential therapeutic target for triple−negative breast cancer. *Cancer Sci.* 111:2440. 10.1111/cas.14498 32437068 PMC7385350

[B95] Mody H. R. Hung S. W. Pathak R. K. Griffin J. Cruz-Monserrate Z. Govindarajan R. (2017). miR-202 diminishes TGFbeta receptors and attenuates TGFbeta1-Induced EMT in pancreatic cancer. *Mol. Cancer Res.* 15 1029–1039. 10.1158/1541-7786.mcr-16-0327 28373289 PMC5540775

[B96] Mu Y. Li N. Cui Y.-L. (2018). The lncRNA CCAT1 upregulates TGFβR1 via sponging miR-490-3p to promote TGFβ1-induced EMT of ovarian cancer cells. *Cancer Cell Int.* 18:145. 10.1186/s12935-018-0604-1 PMC6148998 30250403

[B97] Nie J. Jiang H. C. Zhou Y. C. Jiang B. He W. J. Wang Y. F. (2019). MiR-125b regulates the proliferation and metastasis of triple negative breast cancer cells via the Wnt/beta-catenin pathway and EMT. *Biosci. Biotechnol. Biochem.* 83 1062–1071. 10.1080/09168451.2019.1584521 30950326

[B98] Ning X. Wang C. Zhang M. Wang K. (2019). Ectopic expression of miR-147 inhibits stem cell marker and epithelial-mesenchymal transition (EMT)-Related protein expression in colon cancer cells. *Oncol. Res.* 27 399–406. 10.3727/096504018x15179675206495 29426374 PMC7848281

[B99] Pan J. Fang S. Tian H. Zhou C. Zhao X. Tian H. (2020). lncRNA JPX/miR-33a-5p/Twist1 axis regulates tumorigenesis and metastasis of lung cancer by activating Wnt/β-catenin signaling. *Mol. Cancer* 19 1–17. 31941509 10.1186/s12943-020-1133-9 PMC6961326

[B100] Pan Q. Meng L. Ye J. Wei X. Shang Y. Tian Y. (2017). Transcriptional repression of miR-200 family members by Nanog in colon cancer cells induces epithelial-mesenchymal transition (EMT). *Cancer Lett.* 392 26–38. 10.1016/j.canlet.2017.01.039 28163188

[B101] Park Y. R. Kim S. L. Lee M. R. Seo S. Y. Lee J. H. Kim S. H. (2017). MicroRNA-30a-5p (miR-30a) regulates cell motility and EMT by directly targeting oncogenic TM4SF1 in colorectal cancer. *J. Cancer Res. Clin. Oncol.* 143 1915–1927. 10.1007/s00432-017-2440-4 28528497 PMC11819409

[B102] Qi J. C. Yang Z. Zhang Y. P. Lu B. S. Yin Y. W. Liu K. L. (2019). miR-20b-5p, TGFBR2, and E2F1 form a regulatory loop to participate in epithelial to mesenchymal transition in prostate cancer. *Front. Oncol.* 9:1535. 10.3389/fonc.2019.01535 32010624 PMC6974577

[B103] Qian W. Huang T. Feng W. (2020). Circular RNA HIPK3 promotes EMT of cervical cancer through sponging miR-338-3p to up-regulate HIF-1alpha. *Cancer Manag. Res.* 12 177–187. 10.2147/cmar.s232235 32021434 PMC6957911

[B104] Qian W. Ren Z. Lu X. (2019). Knockdown of long non-coding RNA TUG1 suppresses nasopharyngeal carcinoma progression by inhibiting epithelial-mesenchymal transition (EMT) via the promotion of miR-384. *Biochem. Biophys. Res. Commun.* 509 56–63. 10.1016/j.bbrc.2018.12.011 30581000

[B105] Qin C. Zhao F. (2017). Long non-coding RNA TUG1 can promote proliferation and migration of pancreatic cancer via EMT pathway. *Eur. Rev. Med. Pharmacol. Sci.* 21 2377–2384. 28617552

[B106] Qiu J. J. Lin X. J. Tang X. Y. Zheng T. T. Zhang X. Y. Hua K. Q. (2020). Long noncoding RNA TC0101441 induces epithelial-mesenchymal transition in epithelial ovarian cancer metastasis by downregulating KiSS1. *Int. J. Cancer* 146 2588–2598. 10.1002/ijc.32692 31577838

[B107] Ren D. Yang Q. Dai Y. Guo W. Du H. Song L. (2017). Oncogenic miR-210-3p promotes prostate cancer cell EMT and bone metastasis via NF-kappaB signaling pathway. *Mol. Cancer* 16:117. 10.1186/s12943-017-0688-6 PMC5504657 28693582

[B108] Ren P. Zhang H. Chang L. Hong X. Xing L. (2020). LncRNA NR2F1-AS1 promotes proliferation and metastasis of ESCC cells via regulating EMT. *Eur. Rev. Med. Pharmacol. Sci.* 24 3686–3693. 32329844 10.26355/eurrev_202004_20831

[B109] Ren Y. Huang W. Weng G. Cui P. Liang H. Li Y. (2019). lncrna PVT1 promotes proliferation, invasion and epithelial–mesenchymal transition of renal cell carcinoma cells through downregulation of mir-16-5p. *OncoTargets Ther.* 12:2563. 10.2147/ott.s190239 31040699 PMC6454988

[B110] Roche J. (2018). The epithelial-to-mesenchymal transition in cancer. *Cancers* 10:52. 10.3390/cancers10020052 PMC5836084 29462906

[B111] Rogers T. J. Christenson J. L. Greene L. I. O’Neill K. I. Williams M. M. Gordon M. A. (2019). Reversal of triple-negative breast cancer EMT by miR-200c decreases tryptophan catabolism and a program of immunosuppression. *Mol. Cancer Res.* 17 30–41. 10.1158/1541-7786.mcr-18-0246 30213797 PMC6318067

[B112] Sato H. Shien K. Tomida S. Okayasu K. Suzawa K. Hashida S. (2017). Targeting the miR-200c/LIN28B axis in acquired EGFR-TKI resistance non-small cell lung cancer cells harboring EMT features. *Sci. Rep.* 7:40847. 10.1038/srep40847 PMC5233972 28084458

[B113] Shang Y. Chen H. Ye J. Wei X. Liu S. Wang R. H. I. F. - (2017). 1alpha/Ascl2/miR-200b regulatory feedback circuit modulated the epithelial-mesenchymal transition (EMT) in colorectal cancer cells. *Exp. Cell Res.* 360 243–256. 10.1016/j.yexcr.2017.09.014 28899657

[B114] Shen J. Hong L. Yu D. Cao T. Zhou Z. He S. (2019). LncRNA XIST promotes pancreatic cancer migration, invasion and EMT by sponging miR-429 to modulate ZEB1 expression. *Int. J. Biochem. Cell Biol.* 113 17–26. 10.1016/j.biocel.2019.05.021 31163263

[B115] Shi D. Guo L. Sun X. Shang M. Meng D. Zhou X. (2020). UTMD inhibit EMT of breast cancer through the ROS/miR-200c/ZEB1 axis. *Sci. Rep.* 10:6657. 10.1038/s41598-020-63653-w PMC7170845 32313093

[B116] Shi L. Wang Y. Lu Z. Zhang H. Zhuang N. Wang B. (2017). miR-127 promotes EMT and stem-like traits in lung cancer through a feed-forward regulatory loop. *Oncogene* 36 1631–1643. 10.1038/onc.2016.332 27869168

[B117] Su C. Cheng X. Li Y. Han Y. Song X. Yu D. (2018). MiR-21 improves invasion and migration of drug-resistant lung adenocarcinoma cancer cell and transformation of EMT through targeting HBP1. *Cancer Med.* 7 2485–2503. 10.1002/cam4.1294 29663730 PMC6010699

[B118] Su M. Xiao Y. Ma J. Tang Y. Tian B. Zhang Y. (2019). Circular RNAs in cancer: emerging functions in hallmarks, stemness, resistance and roles as potential biomarkers. *Mol. Cancer* 18 1–17. 10.1016/j.canlet.2020.01.031 30999909 PMC6471953

[B120] Sun J. Li Q. Lian X. Zhu Z. Chen X. Pei W. (2019). MicroRNA-29b mediates lung mesenchymal-epithelial transition and prevents lung fibrosis in the silicosis model. *Mol. Ther. Nucleic Acids* 14 20–31. 10.1016/j.omtn.2018.10.017 30529807 PMC6282658

[B121] Sun Y. Shen S. Liu X. Tang H. Wang Z. Yu Z. (2014). MiR-429 inhibits cells growth and invasion and regulates EMT-related marker genes by targeting Onecut2 in colorectal carcinoma. *Mol. Cell Biochem.* 390 19–30. 10.1007/s11010-013-1950-x 24402783 PMC3972435

[B122] Taheri M. Pouresmaeili F. Omrani M. D. Habibi M. Sarrafzadeh S. Noroozi R. (2017). Association of ANRIL gene polymorphisms with prostate cancer and benign prostatic hyperplasia in an Iranian population. *Biomark. Med.* 11 413–422. 10.2217/bmm-2016-0378 28621612

[B123] Tan K. Huang G. Fang Q. (2017). MiR-486-5p prevents migration, invasion and EMT by regulating smad2 in breast cancer. *Int. J. Clin. Exp. Med.* 10 8942–8949.

[B124] Tang H. Wang X. Zhang M. Yan Y. Huang S. Ji J. (2020). MicroRNA-200b/c-3p regulate epithelial plasticity and inhibit cutaneous wound healing by modulating TGF-β-mediated RAC1 signaling. *Cell Death Dis.* 11 1–17. 33122632 10.1038/s41419-020-03132-2 PMC7596237

[B125] Tao L. Shu-Ling W. Jing-Bo H. Ying Z. Rong H. Xiang-Qun L. (2020). MiR-451a attenuates doxorubicin resistance in lung cancer via suppressing epithelialmesenchymal transition (EMT) through targeting c-Myc. *Biomed Pharmacother.* 125:109962. 10.1016/j.biopha.2020.109962 32106373

[B126] Thiery J. P. Acloque H. Huang R. Y. Nieto M. A. (2009). Epithelial-mesenchymal transitions in development and disease. *Cell* 139 871–890. 10.1016/j.cell.2009.11.007 19945376

[B127] Tian X.-J. Zhang H. Xing J. (2013). Coupled reversible and irreversible bistable switches underlying TGFβ-induced epithelial to mesenchymal transition. *Biophys. J.* 105 1079–1089. 10.1016/j.bpj.2013.07.011 23972859 PMC3752104

[B128] Tong Y. Wang M. Dai Y. Bao D. Zhang J. Pan H. (2019). LncRNA HOXA-AS3 sponges miR-29c to facilitate cell proliferation, metastasis, and EMT process and activate the MEK/ERK signaling pathway in hepatocellular carcinoma. *Hum. Gene Ther. Clin. Dev.* 30 129–141. 10.1089/humc.2018.266 30963785

[B129] Toraman S. Alakus T. B. Turkoglu I. (2020). Convolutional capsnet: a novel artificial neural network approach to detect COVID-19 disease from X-ray images using capsule networks. *Chaos Solitons Fract.* 140:110122. 10.1016/j.chaos.2020.110122 32834634 PMC7357532

[B130] Tuo Z. Zhang J. Xue W. (2018). LncRNA TP73-AS1 predicts the prognosis of bladder cancer patients and functions as a suppressor for bladder cancer by EMT pathway. *Biochem. Biophys. Res. Commun.* 499 875–881. 10.1016/j.bbrc.2018.04.010 29625110

[B131] Wang D. X. Zou Y. J. Zhuang X. B. Chen S. X. Lin Y. Li W. L. (2017). Sulforaphane suppresses EMT and metastasis in human lung cancer through miR-616-5p-mediated GSK3beta/beta-catenin signaling pathways. *Acta Pharmacol. Sin.* 38 241–251. 10.1038/aps.2016.122 27890917 PMC5309754

[B132] Wang G. Fu Y. Liu G. Ye Y. Zhang X. (2017). miR-218 inhibits proliferation, migration, and EMT of gastric cancer cells by targeting WASF3. *Oncol Res.* 25 355–364. 10.3727/096504016x14738114257367 27642088 PMC7841020

[B133] Wang H. Y. Liu Y. N. Wu S. G. Hsu C. L. Chang T. H. Tsai M. F. (2020). MiR-200c-3p suppression is associated with development of acquired resistance to epidermal growth factor receptor (EGFR) tyrosine kinase inhibitors in EGFR mutant non-small cell lung cancer via a mediating epithelial-to-mesenchymal transition (EMT) process. *Cancer Biomark.* 28 351–363. 10.3233/cbm-191119 32417760 PMC12662365

[B134] Wang L. Kang F. B. Wang J. Yang C. He D. W. (2019). Downregulation of miR-205 contributes to epithelial-mesenchymal transition and invasion in triple-negative breast cancer by targeting HMGB1-RAGE signaling pathway. *Anticancer Drugs* 30 225–232. 10.1097/cad.0000000000000705 30334817 PMC6410973

[B135] Wang R. Lu X. Yu R. (2020). lncRNA MALAT1 promotes EMT process and cisplatin resistance of oral squamous cell carcinoma via PI3K/AKT/m-TOR signal pathway. *OncoTargets Ther.* 13:4049. 10.2147/ott.s251518 32494159 PMC7231756

[B136] Wang S. Wang X. Li J. Meng S. Liang Z. Xu X. (2017). c-Met, CREB1 and EGFR are involved in miR-493-5p inhibition of EMT via AKT/GSK-3beta/Snail signaling in prostate cancer. *Oncotarget* 8 82303–82313. 10.18632/oncotarget.19398 29137265 PMC5669891

[B137] Wang S. Wu Y. Yang S. Liu X. Lu Y. Liu F. (2020). miR-874 directly targets AQP3 to inhibit cell proliferation, mobility and EMT in non-small cell lung cancer. *Thorac. Cancer* 11 1550–1558. 10.1111/1759-7714.13428 32301290 PMC7262918

[B138] Wang X. Zhang M. Liu H. (2019). LncRNA17A regulates autophagy and apoptosis of SH-SY5Y cell line as an in vitro model for Alzheimer’s disease. *Biosci. Biotechnol. Biochem.* 83 609–621. 10.1080/09168451.2018.1562874 30652945

[B139] Wang Y. Gu J. Lin X. Yan W. Yang W. Wu G. (2018). lncRNA BANCR promotes EMT in PTC via the Raf/MEK/ERK signaling pathway. *Oncol. Lett.* 15 5865–5870. 29552216 10.3892/ol.2018.8017 PMC5840535

[B140] Wang Y. Wang X. Li Z. Chen L. Zhou L. Li C. (2017). Two single nucleotide polymorphisms (rs2431697 and rs2910164) of miR-146a are associated with risk of coronary artery disease. *Int. J. Environ. Res. Public Health* 14:514. 10.3390/ijerph14050514 28489066 PMC5451965

[B142] Wu B.-Q. Jiang Y. Zhu F. Sun D.-L. He X.-Z. (2017). Long noncoding RNA PVT1 promotes EMT and cell proliferation and migration through downregulating p21 in pancreatic cancer cells. *Technol. Cancer Res. Treat.* 16 819–827. 10.1177/1533034617700559 28355965 PMC5762037

[B143] Wu J. Weng Y. He F. Liang D. Cai L. (2018). LncRNA MALAT-1 competitively regulates miR-124 to promote EMT and development of non-small-cell lung cancer. *Anti Cancer Drugs* 29 628–636. 10.1097/cad.0000000000000626 29782349

[B145] Wu Q. Ma J. Meng W. Hui P. (2020). DLX6-AS1 promotes cell proliferation, migration and EMT of gastric cancer through FUS-regulated MAP4K1. *Cancer Biol. Ther.* 21 17–25. 10.1080/15384047.2019.1647050 31591939 PMC7012178

[B146] Wu S. Wu E. Wang D. Niu Y. Yue H. Zhang D. (2020). LncRNA HRCEG, regulated by HDAC1, inhibits cells proliferation and epithelial-mesenchymal-transition in gastric cancer. *Cancer Genet.* 241 25–33. 10.1016/j.cancergen.2019.12.007 31964588

[B147] Xiang Y. Liao X. H. Yu C. X. Yao A. Qin H. Li J. P. (2017). MiR-93-5p inhibits the EMT of breast cancer cells via targeting MKL-1 and STAT3. *Exp. Cell Res.* 357 135–144. 10.1016/j.yexcr.2017.05.007 28499590

[B148] Xiao C. Wu C. Hu H. (2016). LncRNA UCA1 promotes epithelial-mesenchymal transition (EMT) of breast cancer cells via enhancing Wnt/beta-catenin signaling pathway. *Eur. Rev. Med. Pharmacol. Sci.* 20 2819–2824. 27424981

[B149] Xiong T. Huang C. Li J. Yu S. Chen F. Zhang Z. (2020). LncRNA NRON promotes the proliferation, metastasis and EMT process in bladder cancer. *J. Cancer* 11 1751–1760. 10.7150/jca.37958 32194786 PMC7052857

[B150] Xu D. Dong P. Xiong Y. Yue J. Konno Y. Ihira K. (2020). MicroRNA-361-mediated inhibition of HSP90 expression and EMT in cervical cancer is counteracted by oncogenic lncRNA NEAT1. *Cells* 9:632. 10.3390/cells9030632 32151082 PMC7140536

[B151] Xu D. Liu S. Zhang L. Song L. (2017). MiR-211 inhibits invasion and epithelial-to-mesenchymal transition (EMT) of cervical cancer cells via targeting MUC4. *Biochem. Biophys. Res. Commun.* 485 556–562. 10.1016/j.bbrc.2016.12.020 27923652

[B153] Xu Y. Pan Z. G. Shu L. Li Q. J. (2018). Podocalyxin-like, targeted by miR-138, promotes colorectal cancer cell proliferation, migration, invasion and EMT. *Eur. Rev. Med. Pharmacol. Sci.* 22 8664–8674. 30575907 10.26355/eurrev_201812_16631

[B154] Xuan W. Zhou C. You G. (2020). LncRNA LINC00668 promotes cell proliferation, migration, invasion ability and EMT process in hepatocellular carcinoma by targeting miR-532-5p/YY1 axis. *Biosci. Rep.* 40:BSR20192697. 10.1042/BSR20192697 PMC7214398 32249890

[B155] Xue Y. B. Ding M. Q. Xue L. Luo J. H. (2019). CircAGFG1 sponges miR-203 to promote EMT and metastasis of non-small-cell lung cancer by upregulating ZNF281 expression. *Thorac. Cancer* 10 1692–1701. 10.1111/1759-7714.13131 31243884 PMC6669801

[B156] Yan H. Li H. Silva M. A. Guan Y. Yang L. Zhu L. (2019). LncRNA FLVCR1-AS1 mediates miR-513/YAP1 signaling to promote cell progression, migration, invasion and EMT process in ovarian cancer. *J. Exp. Clin. Cancer Res.* 38 1–13. 31412903 10.1186/s13046-019-1356-z PMC6694549

[B157] Yan K. Tian J. Shi W. Xia H. Zhu Y. (2017). LncRNA SNHG6 is associated with poor prognosis of gastric cancer and promotes cell proliferation and EMT through epigenetically silencing p27 and sponging miR-101-3p. *Cell. Physiol. Biochem.* 42 999–1012. 10.1159/000478682 28683446

[B158] Yang D. Ma M. Zhou W. Yang B. Xiao C. (2017). Inhibition of miR-32 activity promoted EMT induced by PM2.5 exposure through the modulation of the Smad1-mediated signaling pathways in lung cancer cells. *Chemosphere* 184 289–298. 10.1016/j.chemosphere.2017.05.152 28601662

[B160] Yang R. M. Zhan M. Xu S. W. Long M. M. Yang L. H. Chen W. (2017). miR-3656 expression enhances the chemosensitivity of pancreatic cancer to gemcitabine through modulation of the RHOF/EMT axis. *Cell Death Dis.* 8:e3129. 10.1038/cddis.2017.530 29048402 PMC5682692

[B162] Yang X. Hu Y. Liu Y. Liu W. Zhao X. Liu M. (2017). C14orf28 downregulated by miR-519d contributes to oncogenicity and regulates apoptosis and EMT in colorectal cancer. *Mol. Cell Biochem.* 434 197–208. 10.1007/s11010-017-3049-2 28455792

[B163] Yao X. Liu C. Liu C. Xi W. Sun S. Gao Z. (2019). lncRNA SNHG7 sponges miR−425 to promote proliferation, migration, and invasion of hepatic carcinoma cells via Wnt/β−catenin/EMT signalling pathway. *Cell Biochem. Funct.* 37 525–533. 10.1002/cbf.3429 31478234 PMC6851833

[B164] Ye F. Tian L. Zhou Q. Feng D. (2019). LncRNA FER1L4 induces apoptosis and suppresses EMT and the activation of PI3K/AKT pathway in osteosarcoma cells via inhibiting miR-18a-5p to promote SOCS5. *Gene* 721:144093. 10.1016/j.gene.2019.144093 31473323

[B165] Yingping L. Jinglong C. (2019). miR-425 suppresses EMT and the development of TNBC (triple-negative breast cancer) by targeting the TGF-b1/SMAD 3 signaling pathway. *RSC Adv.* 9 151–165. 10.1039/c8ra08872a PMC9059317 35521597

[B166] Yuan C. Luo X. Zhan X. Zeng H. Duan S. E. M. T. (2020). related circular RNA expression profiles identify circSCYL2 as a novel molecule in breast tumor metastasis. *Int. J. Mol. Med.* 45 1697–1710. 32236616 10.3892/ijmm.2020.4550 PMC7169655

[B167] Zeng T. Ni H. Yu Y. Zhang M. Wu M. Wang Q. (2019). BACE1-AS prevents BACE1 mRNA degradation through the sequestration of BACE1-targeting miRNAs. *J. Chem. Neuroanat.* 98 87–96. 10.1016/j.jchemneu.2019.04.001 30959172

[B168] Zhai W. Li S. Zhang J. Chen Y. Ma J. Kong W. (2018). Sunitinib-suppressed miR-452-5p facilitates renal cancer cell invasion and metastasis through modulating SMAD4/SMAD7 signals. *Mol. Cancer* 17:157. 10.1186/s12943-018-0906-x PMC6231268 30419914

[B169] Zhang D.-M. Lin Z.-Y. Yang Z.-H. Wang Y.-Y. Wan D. Zhong J.-L. (2017a). IncRNA H19 promotes tongue squamous cell carcinoma progression through β-catenin/GSK3β/EMT signaling via association with EZH2. *Am. J. Transl. Res.* 9:3474. PMC5527262 28804564

[B170] Zhang K. Chen J. Song H. Chen L.-B. (2017b). SNHG16/miR-140-5p axis promotes esophagus cancer cell proliferation, migration and EMT formation through regulating ZEB1. *Oncotarget* 9 1028–1040. 10.18632/oncotarget.23178 29416674 PMC5787416

[B171] Zhang K. Li X. Y. Wang Z. M. Han Z. F. Zhao Y. H. (2017c). MiR-22 inhibits lung cancer cell EMT and invasion through targeting Snail. *Eur. Rev. Med. Pharmacol. Sci.* 21 3598–3604. 28925484

[B172] Zhang L. Liu X. L. Yuan Z. Cui J. Zhang H. (2019). MiR-99a suppressed cell proliferation and invasion by directly targeting HOXA1 through regulation of the AKT/mTOR signaling pathway and EMT in ovarian cancer. *Eur. Rev. Med. Pharmacol. Sci.* 23 4663–4672. 31210292 10.26355/eurrev_201906_18046

[B173] Zhang L. Y. Chen Y. Jia J. Zhu X. He Y. Wu L. M. (2019). MiR-27a promotes EMT in ovarian cancer through active Wnt/-catenin signalling by targeting FOXO1. *Cancer Biomark.* 24 31–42. 10.3233/cbm-181229 30614794 PMC13082501

[B174] Zhang M. Duan W. Sun W. (2019). LncRNA SNHG6 promotes the migration, invasion, and epithelial-mesenchymal transition of colorectal cancer cells by miR-26a/EZH2 axis. *OncoTargets Ther.* 12:3349. 10.2147/ott.s197433 31118686 PMC6504670

[B175] Zhang P. Tang W. M. Zhang H. Li Y. Q. Peng Y. Wang J. (2017d). MiR-646 inhibited cell proliferation and EMT-induced metastasis by targeting FOXK1 in gastric cancer. *Br. J. Cancer* 117 525–534. 10.1038/bjc.2017.181 28632723 PMC5558677

[B176] Zhang P. F. Wang F. Wu J. Wu Y. Huang W. Liu D. (2019). LncRNA SNHG3 induces EMT and sorafenib resistance by modulating the miR−128/CD151 pathway in hepatocellular carcinoma. *J. Cell. Physiol.* 234 2788–2794. 10.1002/jcp.27095 30132868

[B177] Zhang S. Xiao J. Chai Y. Yan Du Y Liu Z. Huang K. (2017e). LncRNA-CCAT1 promotes migration, invasion, and EMT in intrahepatic cholangiocarcinoma through suppressing miR-152. *Digest. Dis. Sci.* 62 3050–3058. 10.1007/s10620-017-4759-8 28921383

[B178] Zhang T. Pang P. Fang Z. Guo Y. Li H. Li X. (2018a). Expression of BC1 impairs spatial learning and memory in Alzheimer’s disease via APP translation. *Mol. Neurobiol.* 55 6007–6020. 10.1007/s12035-017-0820-z 29134514

[B179] Zhang W. Yuan W. Song J. Wang S. Gu X. (2018b). LncRNA CPS1-IT1 suppresses EMT and metastasis of colorectal cancer by inhibiting hypoxia-induced autophagy through inactivation of HIF-1α. *Biochimie* 144 21–27. 10.1016/j.biochi.2017.10.002 29017924

[B180] Zhang W. Zhai Y. Wang W. Cao M. Ma C. (2018c). Enhanced expression of lncRNA TP73-AS1 predicts unfavorable prognosis for gastric cancer and promotes cell migration and invasion by induction of EMT. *Gene* 678 377–383. 10.1016/j.gene.2018.08.055 30118890

[B181] Zhang X. Zhang Y. Mao Y. Ma X. (2018d). The lncRNA PCAT1 is correlated with poor prognosis and promotes cell proliferation, invasion, migration and EMT in osteosarcoma. *Onco Targets Ther.* 11 629–638. 10.2147/ott.s152063 29430187 PMC5797453

[B182] Zhang Y. Li B. Zhang B. Ma P. Wu Q. Ming L. (2018e). LncRNA SBF2-AS1 promotes hepatocellular carcinoma metastasis by regulating EMT and predicts unfavorable prognosis. *Eur. Rev. Med. Pharmacol. Sci.* 22 6333–6341. 30338801 10.26355/eurrev_201810_16044

[B183] Zhang Y. Yuan Y. Zhang Y. Cheng L. Zhou X. Chen K. (2020). SNHG7 accelerates cell migration and invasion through regulating miR-34a-Snail-EMT axis in gastric cancer. *Cell Cycle* 19 142–152. 10.1080/15384101.2019.1699753 31814518 PMC6927713

[B184] Zhang Z. Che X. Yang N. Bai Z. Wu Y. Zhao L. (2017). miR-135b-5p Promotes migration, invasion and EMT of pancreatic cancer cells by targeting NR3C2. *Biomed Pharmacother.* 96 1341–1348. 10.1016/j.biopha.2017.11.074 29196101

[B185] Zhang Z. Yang Y. Zhang X. (2017). MiR-770 inhibits tumorigenesis and EMT by targeting JMJD6 and regulating WNT/beta-catenin pathway in non-small cell lung cancer. *Life Sci.* 188 163–171. 10.1016/j.lfs.2017.09.002 28882645

[B186] Zhao H. Diao C. Wang X. Xie Y. Liu Y. Gao X. (2018). LncRNA BDNF-AS inhibits proliferation, migration, invasion and EMT in oesophageal cancer cells by targeting miR-214. *J. Cell. Mol. Med.* 22 3729–3739. 10.1111/jcmm.13558 29896888 PMC6050505

[B187] Zhao L. Sun H. Kong H. Chen Z. Chen B. Zhou M. (2017). The Lncrna-TUG1/EZH2 axis promotes pancreatic cancer cell proliferation, migration and EMT phenotype formation through sponging Mir-382. *Cell. Physiol. Biochem.* 42 2145–2158. 10.1159/000479990 28813705

[B188] Zhao S. Luo L. Xiang Q. Zhu Z. Wang J. Liu Y. (2019). Cancer-derived exosomal miR-199b-5p inhibits distant metastases of prostate cancer by counteracting the DDR1-MAPK/ERK-EMT pathway. *EBioMedicine* [Preprint]. 10.2139/ssrn.3475571

[B189] Zheng F. Li J. Ma C. Tang X. Tang Q. Wu J. (2020). Novel regulation of miR-34a-5p and HOTAIR by the combination of berberine and gefitinib leading to inhibition of EMT in human lung cancer. *J. Cell. Mol. Med.* 24 5578–5592. 10.1111/jcmm.15214 32248643 PMC7214156

[B190] Zhou J. Zou L. Zhu T. (2020). Long non-coding RNA LINC00665 promotes metastasis of breast cancer cells by triggering EMT. *Eur. Rev. Med. Pharmacol. Sci.* 24 3097–3104. 32271427 10.26355/eurrev_202003_20674

[B191] Zhou W. Ye X. L. Xu J. Cao M. G. Fang Z. Y. Li L. Y. (2017). The lncRNA H19 mediates breast cancer cell plasticity during EMT and MET plasticity by differentially sponging miR-200b/c and let-7b. *Sci. Signal.* 10:eaak9557. 10.1126/scisignal.aak9557 28611183

[B193] Zhu L. Yang N. Du G. Li C. Liu G. Liu S. (2018). LncRNA CRNDE promotes the epithelial-mesenchymal transition of hepatocellular carcinoma cells via enhancing the Wnt/β-catenin signaling pathway. *J. Cell. Biochem.* 120 1156–1164. 10.1002/jcb.26762 30430650 PMC6587876

[B194] Zoi P. Marco F. Christoph R. Martin G. Nikos K. K. (2020). miR-200b restrains EMT and aggressiveness and regulates matrix composition depending on ER status and signaling in mammary cancer. *Matrix Biol. Plus* 6-7 100024. 10.1016/j.mbplus.2020.100024 33543022 PMC7852204

