# Corrigendum: The impact of non-coding RNAs in the epithelial to mesenchymal transition

**DOI:** 10.3389/fmolb.2024.1524742

**Published:** 2024-12-11

**Authors:** Bashdar Mahmud Hussen, Hamed Shoorei, Mahdi Mohaqiq, Marcel E. Dinger, Hazha Jamal Hidayat, Mohammad Taheri, Soudeh Ghafouri-Fard

**Affiliations:** ^1^ Pharmacognosy Department, College of Pharmacy, Hawler Medical University, Erbil, Iraq; ^2^ Department of Anatomical Sciences, Faculty of Medicine, Birjand University of Medical Sciences, Birjand, Iran; ^3^ Wake Forest Institute for Regenerative Medicine, School of Medicine, Wake Forest University, Winston-Salem, NC, United States; ^4^ School of Biotechnology and Biomolecular Sciences, University of New South Wales, Sydney, NSW, Australia; ^5^ Department of Biology, College of Education, Salahaddin University-Erbil, Erbil, Iraq; ^6^ Urology and Nephrology Research Center, Shahid Beheshti University of Medical Sciences, Tehran, Iraq; ^7^ Department of Medical Genetics, Shahid Beheshti University of Medical Sciences, Tehran, Iraq

**Keywords:** lncRNA, miRNA, epithelial to mesenchymal transition, expression, biomarker

In the published article, the References column was incorrect in **Supplementary Table 1**. The table has been updated in the original article supplementary material.

In the published article, **Supplementary Table 2** was mistakenly not included in the publication. The table has been added to the original article supplementary material.

After publication of this article, it has come to the authors’ attention that the following referenced articles have been retracted. These references have been removed from the original article.

Chong L., Lingling S., Jiaying S. (2019). Circular RNA hsa_circ_0000467 modulates SGK1 to facilitate cell migration, metastasis, and EMT while repressing apoptosis in colorectal cancer by sponging miR-383-5p. *RSC Adv.* 9 39294–39303. 10.1039/c9ra07900a.

Fan H., Liu X., Zheng W. W., Zhuang Z. H., Wang C. D. (2017). MiR-150 alleviates EMT and cell invasion of colorectal cancer through targeting Gli1. *Eur. Rev. Med. Pharmacol. Sci.* 21 4853–4859.

Jin D., Guo J., Wu Y., Du J., Wang X., An J., et al. (2019). UBE2C, directly targeted by miR-548e-5p, increases the cellular growth and invasive abilities of cancer cells interacting with the EMT marker protein zinc finger E-box binding homeobox 1/2 in NSCLC. *Theranostics* 9 2036–2055. 10.7150/thno.32738.

Li R., Liu J., Qi J. (2020a). Knockdown of long non-coding RNA CCAT1 suppresses proliferation and EMT of human cervical cancer cell lines by down-regulating Runx2. *Exp. Mol. Pathol.* 113:104380. 10.1016/j.yexmp.2020.104380.

Li X., Wang S.-W., Xi-Ling L., Yu F.-Y., Cong H.-M. (2020c). Knockdown of long non-coding RNA TUG1 depresses apoptosis of hippocampal neurons in Alzheimer’s disease by elevating microRNA-15a and repressing ROCK1 expression. *Inflamm. Res.* 69 897–910. 10.1007/s00011-020-01364-8.

Li Z.-T., Zhang X., Wang D.-W., Xu J., Kou K.-J., Wang Z.-W., et al. (2020d). Overexpressed lncRNA GATA6-AS1 inhibits LNM and EMT via FZD4 through the Wnt/β-catenin signaling pathway in GC. *Mol. Ther. Nucleic Acids* 19 827–840. 10.1016/j.omtn.2019.09.034.

Lin X., Wang S., Sun M., Zhang C., Wei C., Yang C., et al. (2019). miR-195-5p/NOTCH2-mediated EMT modulates IL-4 secretion in colorectal cancer to affect M2-like TAM polarization. *J. Hematol. Oncol.* 12:20.

Liu S., Song L., Yao H., Zhang L., Xu D., Gao F., et al. (2016). MiR-375 is epigenetically downregulated by HPV-16 E6 mediated DNMT1 upregulation and modulates EMT of cervical cancer cells by suppressing lncRNA MALAT1. *PLoS One* 11:e0163460.

Liu W., Yang Y. J., An Q. (2020). LINC00963 promotes ovarian cancer proliferation, migration and EMT via the miR-378g/CHI3L1 Axis. *Cancer Manag. Res.* 12 463–473. 10.2147/cmar.s229083.

Sun J., Hu J., Wang G., Yang Z., Zhao C., Zhang X., et al. (2018). LncRNA TUG1 promoted KIAA1199 expression via miR-600 to accelerate cell metastasis and epithelial-mesenchymal transition in colorectal cancer. *J. Exp. Clin. Cancer Res.* 37:106.

Wang Z., Wang J., Wang K., Zhou Y., Wang J. (2020). LncRNA FEZF1-AS1 promoted chemoresistance, autophagy and epithelial-mesenchymal transition (EMT) through regulation of miR-25-3p/ITGB8 axis in prostate cancer. *Eur. Rev. Med. Pharmacol. Sci.* 24 2281–2293.

Wu K., Li L., Li L., Wang D. (2020). Long non-coding RNA HAL suppresses the migration and invasion of serous ovarian cancer by inhibiting EMT signaling pathway. *Biosci. Rep*. 40:BSR20194496.

Xu W., Sun X., Zang C., Jiang Y. (2020). lncRNA SNHG7 promotes tumorigenesis of nasopharyngeal carcinoma via epithelial-to-mesenchymal transition. *Oncol. Lett.* 19 2721–2726.

Yang F., Shao C., Wei K., Jing X., Qin Z., Shi Y., et al. (2019). miR-942 promotes tumor migration, invasion, and angiogenesis by regulating EMT via BARX2 in non-small-cell lung cancer. *J. Cell Physiol.* 234 23596–23607. 10.1002/jcp.28928.

Yang T., He X., Chen A., Tan K., Du X. (2018). LncRNA HOTAIR contributes to the malignancy of hepatocellular carcinoma by enhancing epithelial-mesenchymal transition via sponging miR-23b-3p from ZEB1. *Gene* 670 114–122. 10.1016/j.gene.2018.05.06.

Zhou X., Men X., Zhao R., Han J., Fan Z., Wang Y., et al. (2018). miR-200c inhibits TGF-beta-induced-EMT to restore trastuzumab sensitivity by targeting ZEB1 and ZEB2 in gastric cancer. *Cancer Gene Ther.* 25 68–76. 10.1038/s41417-017-0005-y.

A correction has been made to **Introduction**, *miRNAs in EMT*, Paragraph 1. This sentence previously stated:

“In this type of cancer, miR-145-5p, miR-383-5p, miR-3622a-3p, miR-205 and miR-200b inhibit EMT process through targeting CDCA3, SGK1, SALL4, MDM4 and HIF-1α, respectively (Shang et al., 2017; Chong et al., 2019; Chang et al., 2020; Chen et al., 2020; Fan and Wang, 2020).”

The corrected sentence appears below:

“In colorectal cancer, miR-145-5p, miR-3622a-3p, miR-205 and miR-200b inhibit EMT through targeting CDCA3, SALL4, MDM4 and HIF-1α, respectively (Shang et al., 2017; Chang et al., 2020; Chen et al., 2020a; Fan and Wang 2020).”

A correction has been made to **Introduction,** *lncRNAs in EMT*, Paragraph 1. The section previously included the following sentences:

“CCAT1 is an oncogenic lncRNA in cervical cancer cells whose silencing has blocked proliferation, migratory potential, invasiveness and EMT process in these cells. CCAT1 silencing has led to downregulation of Runx2 and suppression of PI3K/AKT signaling in cervical cancer cells PI3K/AKT signal (Li et al., 2020a). HAL is a downregulated lncRNA in serous ovarian cancer tissues and cells. Upregulation of HAL has suppressed invasive potential of these cells and enhanced their apoptosis. HAL has been shown to directly suppress expression of TWIST1. Functional studies has highlighted the role of HAL in regulation of EMT (Wu K. et al., 2020). In ovarian cancer, LINC00963, TC0101441, CCAT1 and PTAR promote EMT through modulation of miR-378g, KiSS1, miR-490-3p and miR-101-3p, respectively (Liang et al., 2018; Mu et al., 2018; Liu et al., 2020; Qiu et al., 2020).”

The corrected sentence appears below:

“In ovarian cancer, TC0101441, CCAT1 and PTAR promote EMT through modulation of KiSS1, miR-490-3p and miR-101-3p, respectively (Liang et al., 2018; Mu et al., 2018; Qiu et al., 2020).”

A correction has been made to **Introduction**, *lncRNAs in EMT*, Paragraph 2. This sentence previously stated:

“Over-expression of LINC00963, FLVCR1-AS1 and LINC00261 has been associated with poor overall survival rate of patients with neoplasm (Yan et al., 2019; Gao et al., 2020; Liu et al., 2020).”

The corrected sentence appears below:

“Over-expression of FLVCR1-AS1 and LINC00261 has been associated with poor overall survival rate of patients with neoplasm (Yan et al., 2019; Gao et al., 2020).”

In the published article, there was an error in Tables 1, 2 as published. These tables cited four retracted articles that should be removed (Lin et al., 2019; Jin et al., 2019; Liu et al., 2020; Sun et al., 2018). The corrected Tables 1, 2 and their captions appear below.

**TABLE 1:** Prognostic roles of EMT-associated miRNAs in cancer (ACT: adjacent control tissue).

Sample number	Kaplan-Meier analysis	References
70 pairs of LC and ACTs	High miR-200c-3p expression was linked with longer survival	Wang H. Y. et al. (2020)
179 pairs of LC and ACTs	High expression of miR-616-5p was linked with poor overall survival	Shi et al. (2017)
49 pairs of LC and ACTs	Decreased miR-874 expression was linked with poor prognosis	Wang S. et al. (2020)
47 pairs of OC and ACTs	Decreased miR-99a expression in was linked with poor prognosis	Zhang L. et al. (2019)
51 pairs of BC and ACTs	Decreased miR-92b expression was linked with poor prognosis	Li Y. Y. et al. (2019)
60 pairs of BC and ACTs	Decreased miR-516a-3p expression was linked with poor prognosis	Chi et al. (2019)
117 pairs of BLC and ACTs	Decreased miR-221expression was linked with poor prognosis	Li F. et al. (2019)
300 pairs of CRC and ACTs	Decreased miR-330 expression was linked with poor prognosis	Mansoori et al. (2020)
80 pairs of CRC and ACTs	High expression of miR-3622a-3p was linked with better overall survival	Chang et al. (2020)
4 pairs of CRC and ACTs	Decreased miR-598 expression was linked with poor prognosis	Wang Y. et al. (2017)
93 pairs of BC and ACTs	Higher expression of miR-365-3p was linked with better overall survival	Gao and Tian (2020)
Breast cancer	Higher expression of miR-335 was linked with poor overall survival	Chen et al. (2019)
157 pairs of PaC and ACTs	Decreased miR-3656 expression was linked with poor prognosis	Yang R. M. et al. (2017)
36 OC tissues and 14 normal ovarian tissue	Decreased miR-195-5p expression was linked with poor prognosis	Dong S. et al. (2019)
35 pairs of GC and ACTs	Decreased miR-125a-5p expression was linked with poor prognosis	Wang X. et al. (2019)
52 pairs of CC and ACTs	Decreased miR-31-3p expression was linked with poor prognosis	Jing et al. (2019)
20 pairs of PCa and ACTs	Decreased miR-33a-5p expression was linked with poor prognosis	Dai Y. et al. (2019)
30 pairs of PCa and ACTs	High expression of miR-199b-5p was linked with poor prognosis	Zhao et al. (2019)
52 pairs of PCa and ACTs	High expression of miR-210-3p was linked with poor prognosis	Ren et al. (2017)
60 pairs of OC and ACTs	High expression of miR-1228 was linked with poor prognosis	Du L. et al. (2020)
36 pairs of tumor specimens and adjacent normal specimens	High expression of miR-127 was linked with poor prognosis	Shi et al. (2017)
20 pairs of RCC and ACTs	High expression of miR-452-5p was linked with poor prognosis	Zhai et al. (2018)
36 pairs of GBC and ACTs	Decreased miR-143-5p expression was linked with poor prognosis	Taheri et al. (2017)

**TABLE 2:** Diagnostic and prognostic role of EMT-associated lncRNAs in cancer (ACTs: adjacent control tissues, OS: overall survival).

Sample number	Area under curve	Sensitivity	Specificity	Kaplan-Meier analysis	Multivariate cox regression	References
50 pairs of SOC and ACTs	—	—	—	High expression of FLVCR1-AS1 was linked with poor OS	High expression of FLVCR1-AS1 was associated with lymphatic metastasis and distant metastasis	Yan et al. (2019)
50 pairs of CCA and ACTs	—	—	—	High expression of LINC00261 was linked with poor OS	High expression of LINC00261 was associated with large tumor size, positive lymph node metastasis, advanced TNM stages, and higher postoperative recurrence	Gao et al. (2020)
76 pairs of GC and ACTs	—	—	—	High expression of TP73-AS1 was linked with poor OS	High expression of TP73-AS1 was associated with depth of invasion and TNM stages	Zhang et al. (2018c)
18 pairs of GC and ACTs	—	—	—	Low expression of HRCEG was linked with poor OS	—	Wu Q. et al. (2020)
162 pairs of GC and ACTs	—	—	—	High expression of SNHG7 was linked with poor OS	High expression of SNHG7 was associated with TNM stage, depth of invasion, lymph-node metastasis, and distant metastasis	Wu S. et al. (2020)
84 pairs of GC and ACTs	—	—	—	High expression of HCP5 was linked with poor OS	High expression of HCP5 was associated with the size of the tumor, lymph nodes metastasize, and the severity of the disease	Zhang et al. (2020)
78 pairs of GC and ACTs	—	—	—	High expression of SNHG6 was linked with poor OS	High expression of SNHG6 was associated with invasion depth, lymph node metastasis, distant metastasis, and TNM stage	Yan et al. (2017)
92 pairs of CRC and ACTs	—	—	—	High expression of HIF1A-AS2 was linked with poor OS	High expression of HIF1A-AS2 was associated with TNM stages	Lin et al. (2018)
338 pairs of CRC and ACTs	—	—	—	High expression of SNHG1 was linked with poor OS	—	Bai et al. (2020)
124 pairs of CRC and ACTs	—	—	—	High expression of PANDAR was linked with poor OS	High expression of PANDAR was associated with tumor diameter, histological differentiation, TNM stage, lymph node metastasis, depth of invasion	Lu et al. (2017)
82 pairs of BC and ACTs	—	—	—	High expression of TP73-AS1 was linked with poor OS	—	Ding et al. (2019)
TCGA database	—	—	—	High expression of PVT1 was linked with poor OS	—	Chang et al. (2018)
40 pairs of HC and ACTs	—	—	—	High expression of SNHG7 was linked with poor OS	—	Yao et al. (2019)
134 pairs of HCC and ACTs	—	—	—	High expression of SBF2-AS1 was linked with poor OS	High expression of SBF2-AS1was associated with vein invasion and TNM stage	Zhang et al. (2018e)
54 pairs of HCC and ACTs	—	—	—	High expression of LOC105372579 was linked with poor OS	High expression of LOC105372579 was associated with tumor size and TNM stage	Changyong et al. (2019)
HCC tissues (n = 38), normal liver tissues (n = 21)	—	—	—	High expression of HULC was linked with poor OS	High expression of HULC was associated with clinical stage and intrahepatic metastases	Li et al. (2016)
76 pairs of HCC and ACTs	—	—	—	High expression of HOXA-AS3 was linked with poor OS	—	Tong et al. (2019)
76 pairs of OSCC and ACTs	—	—	—	High expression of ADAMTS9-AS2 was linked with poor OS	High expression of ADAMTS9-AS2 was associated with tumor size, clinical stage, and lymph node metastasis	Li Y. et al. (2019)
123 OSCC tissues and 50 adjacent non-tumor tissues	—	—	—	High expression of H19 was linked with poor OS	—	Zhang et al. (2017a)
128 pairs of BLC and ACTs	—	—	—	High expression of TP73-AS1 was linked with poor OS and PSF rates	—	Tuo et al. (2018)
48 pairs of NPC and ACTs	—	—	—	High expression of TUG1 was linked with poor OS	—	Qian et al. (2019)
42 pairs of BLC and ACTs	—	—	—	High expression of NRON was linked with poor OS	High expression of NRON was associated with tumor invasion depth	Xiong et al. (2020)
30 pairs of OS and ACTs	—	—	—	High expression of PCAT1 was linked with poor OS.	High expression of PCAT1 was associated with advanced clinical-stage and tumor metastasis	Zhang et al. (2018d)
305 pairs of LUAD and ACTs	—	—	—	High expression of H19 was linked with poor OS	High expression of H19 was associated with tumor diameter and TNM stage	Liu et al. (2019)
107 pairs of LUAD and ACTs	—	—	—	High expression of TTN-AS1 was linked with poor OS	High expression of TTN-AS1 was associated with TNM stage and lymph node involvement	Jia et al. (2019)
50 pairs of NSCLC and ACTs	—	—	—	Low expression of NBR2 was linked with poor OS rate	—	Gao et al. (2019)
86 pairs of NSCLC and ACTs	—	—	—	High expression of FEZF1-AS1 was linked with poor OS	High expression of FEZF1-AS1 was associated with lymph node metastasis, poor differentiation grade, and advanced TNM stage	He et al. (2017)
55 pairs of ESCC and ACTs	0.858	69.7%	91.3%	Low expression of GHET1 was linked with poor OS	High expression of GHET1 was associated with lymph node metastasis, differentiation, and TNM stage	Liu H. et al. (2017)
25 pairs of RCC and ACTs	—	—	—	High expression of PVT1 was linked with poor OS	High expression of PVT1 was associated with TNM stage, fuhrman grade, lymph node involvement, and tumor dimension	Ren et al. (2019)

The authors apologize for these errors and state that these do not change the scientific conclusions of the article in any way. The original article has been updated.

