# Supplementary material for: The Impact of Non-coding RNAs in the Epithelial to Mesenchymal Transition
Source: Front Mol Biosci. 2021 Mar 26;8:665199. doi: 10.3389/fmolb.2021.665199 (PMC8033041; doi:10.3389/fmolb.2021.665199)
Supplement: Supplementary file 1 [file Table_1.docx]

Supplementary Table 1. Function of miRNAs in epithelial-mesenchymal transition (ACTs: adjacent control tissues).

| **Cancer type** | **microRNA** | **Clinical samples** | **Cell line** | **Targets/ Regulators** | **Signaling Pathways** | **Function** | **References** |
| --- | --- | --- | --- | --- | --- | --- | --- |
| Lung Cancer (LC) | miR-451a | - | A549, PC9, H1299, H460 | c-Myc, N-cadherin, Vimentin, E-cadherin | - | miR-451a could reduce EMT through targeting c-Myc in LC. | (Tao et al., 2020) |
| LC | miR-21 | 38 pairs of LC and ACTs | A549, ASTC-a-1, H1299, H322, H441, A549 | PTEN, GSK3β | Akt | miR-21 could promote LC cell EMT via regulation of Akt/GSK3β. | (Dai et al., 2019a) |
| LC | miR-203 | 20 pairs of LC and ACTs | A549, H1299 | ZNF281, E-cadherin, Vimentin | - | miR-203 promotes EMT and metastasis of NSCLC by upregulation ZNF281 expression. | (Xue et al., 2019) |
| LC | miR-590-5p | 33 pairs of LC and ACTs | A549, H1299 | SOX2, E-cadherin, Snail, Slug | - | miR-590-5p could inhibit EMT in LC by targeting SOX2. | (Chang 2019) |
| LC | miR-874 | 49 pairs of LC and ACTs | A549, H1299, BEAS-2B | AQP3, E-cadherin, N-cadherin, Vimentin | PI3K/Akt | miR-874 by targeting AQP3 could inhibit cell proliferation, mobility and EMT in NSCLC. | (Wang et al., 2020b) |
| LC | miR-770 | 50 pairs of LC and ACTs | H522, H650, H1155, H1299, A549 | JMJD6, E-cadherin, Vimentin | Wnt/β-catenin | miR-770 inhibits tumorigenesis and EMT. | (Zhang et al., 2017e) |
| LC | miR-33a | - | NCI-H1299, A549, CRL-9482 | RASSF1C, ABCA1 | - | miR-33a could promote EMT by targeting ABCA1 in LC. | (Amaar and Reeves 2019) |
| LC | miR-200c-3p | 70 pairs of LC and ACTs | PC9, HCC287, HCC4006, H1975, gefitinib-resistant cells | EGFR, E-cadherin, N-cadherin | - | miR-200c-3p could inhibit the EMT process in LC. | (Wang et al., 2020a) |
| LC | miR-34a-5p | - | A549, H1975 | E-cadherin, Vimentin, Snail | - | miR-34a-5p could inhibit EMT in LC. | (Zheng et al., 2020) |
| LC | miR-32 | - | A549, H292 | Smad1, E-cadherin, Vimentin | - | Downregulation of miR-32 could promote EMT through affecting Smad1-associated signaling pathways in LC cells. | (Yang et al., 2017a) |
| LC | miR-127 | 36 pairs of LC and ACTs | PC9, H522, HCC827, H1299, A549 | TNFAIP3, E-cadherin, N-cadherin | NF-κB | Overexpression of miR-127 cancer by targeting TNFAIP3 could induce EMT and promotes cellular migration and invasion in LC. | (Shi et al., 2017) |
| LC | miR-616-5p | 179 pairs of LC and ACTs | 95D, H1299, 95C | GSK3β, β-catenin, E-cadherin, N-cadherin, Vimentin | β-catenin | miR-616-5p silencing inhibits EMT in LC. | (Wang et al., 2017a) |
| LC | miR-21 | - | A549 | HBP1, E-cadherin, Vimentin, snail, slug, β-catenin | HBP1/EMT | miR­21 by targeting HBP1 could promote the viability and EMT of lung adenocarcinoma (LUAD) cancer cells. | (Su et al., 2018) |
| LC | miR-34a | TCPA datasets | A549, H520, BEAS2B | PAI-1, PIAS3, E-cadherin, N-cadherin, Vimentin | STAT3 | miR-34a could promote EMT-mediated metastasis via STAT3. | (Lin et al., 2017) |
| LC | miR-625-3p | - | HCC827, 293T, HCC827GR, HCC827, PC9, PC9GR | AXL, ZEB1, Snail, MMP2, MMP9, N-cadherin, E-cadherin | TGF‑β/Smad | miR-625-3p could inhibit TGF-β1-induced EMT by targeting AXL in LC. | (Du et al., 2020) |
| LC | miR-22 | - | Anip973, AGYZ83-a, MRC-5 | Snail, E-cadherin, N-cadherin | - | miR-22 suppresses LC cell EMT and invasion by inhibiting Snail. | (Zhang et al., 2017b) |
| LC | miR-1-3p, miR-206 | - | PC-9, HCC827 | c-Met, E-cadherin, Vimentin, Snail | Akt/Erk | miR-1-3p/miR-206 could inhibit EMT in LC cells by targeting c-Met. | (Jiao et al., 2018) |
| LC | miR-200c | 3 pairs of LC and ACTs | HBEC-5KT, 293T, PC-9, A549, H3255, | LIN28B, E-cadherin, N-cadherin, Vimentin, | TGF-β/IL6 | Downregulation of miR-200c could inhibit EMT in NSCLC. | (Sato et al., 2017) |
| Prostate Cancer (PCa) | miR-200c | 25 pairs of PCa and ACTs | PC3 | ZEB1, Slug, Vimentin, N-cadherin, β-catenin | - | Downregulation of miR-200c by targeting Zeb1/Slug axis could promote EMT in PCa. | (Basu et al., 2020) |
| PCa | miR-210-3p | 52 pairs of PCa and ACTs | 22RV1, PC-3, VCaP, DU145, LNCaP, RWPE-1 | TNIP1, SOCS1, Vimentin, Fibronectin, E-cadherin | NF-κB | miR-210-3p could promote EMT, invasion and migration. | (Ren et al., 2017) |
| PCa | miR-20b-5p | - | PC3, LNCaP, DU145, VCaP,, 22RV1, RWPE-1 | TGFBR2, E2F1 , E-cadherin, Vimentin, ZO-1, N-cadherin | TGF-β1 | miR-20b-5p could inhibit TGF-β1-induced EMT. | (Qi et al., 2019) |
| PCa | miR-181a | 21 pairs of PCa and ACTs | HEK-293T, PC3, DU145 | MIIP, KLF17, E-cadherin, N-cadherin | - | miR-181a/b-5p could promote PCa cell proliferation, invasion, and EMT. | (Hu et al., 2020) |
| PCa | miR-199b-5p | 30 pairs of PCa and ACTs | RWPE-1,LNCaP, DU145, PC3, VCaP, 22RV1, C4-2, 293T | DDR1, Vimentin, Fibronectin, E-cadherin | MAPK/ERK | Downregulation of miR-199b-5p could promote EMT by activation of MAPK/ERK signaling in PCa. | (Zhao et al., 2021) |
| PCa | miR-33a-5p | 20 pairs of PCa and ACTs | 22RV1, PC-3, VCaP, DU145, LNCaP, RWPE-1, C4-2B | E-cadherin, Vimentin, Fibronectin, ZEB1 | TGF-β | Overexpression of miR-33a-5p by targeting ZEB1/TGF-β axis inhibits EMT, invasion, and migration in PCa. | (Dai et al., 2019b) |
| PCa | miR-493-5p | 59 PCa blood samples and 69 normal control blood samples | PC-3, Du-145, RWPE-1 | c-Met, CREB1, EGFR, E-cadherin, N-cadherin, Fibronectin, Vimentin, Snail | AKT/GSK-3β/Snail | miR-493-5p suppresses EMT via AKT/GSK-3β/Snail axis in PCa. | (Wang et al., 2017c) |
| Ovarian Cancer (OC) | miR-1228 | 60 pairs of OC and ACTs | A2780, OV2008, SKOV3, IGROV1, ES-2 | E-cadherin, N-cadherin | p53 | miR-1228 could inhibit EMT in OC cells. | (Li et al., 2020) |
| OC | miR-27a | 37 pairs of OC and ACTs | OV90, HEK-29, HOSEpiC, HO8910 | FOXO1, E-cadherin, N-cadherin, Vimentin | Wnt/β-catenin | miR-27a could promote EMT in OC via activating Wnt/β-catenin by targeting FOXO1. | (Zhang et al., 2019b) |
| OC | miR-99a | 47 pairs of OC and ACTs | IGROV-1, HO-8910, IOSE80 | HOXA1, E-cadherin, N-cadherin, Vimentin | Akt/mTOR | miR-99a decreases cell proliferation by targeting HOXA1 via regulating Akt/mTOR signaling pathway and EMT in OC. | (Zhang et al., 2019a) |
| OC | miR-101 | - | SKOV3, A2780 | PTAL, FN1, ZO-1, E-cadherin, N-cadherin | - | miR-101 could inhibit EMT and cell migration in OC cells by targeting FN1. | (Liang et al., 2020) |
| OC | miR-186-5p | 36 pairs of OC and ACTs | A2780, SKOV3, IOSE80 | HOXD-AS1, E-cadherin, Vimentin | PIK3R3 | Silencing of miR-186-5p could promote EMT in OC by targeting HOXD-AS1. | (Dong et al., 2019) |
| OC | miR-125a-5p | 40 pairs of OC and ACTs | SKOV3 | TAZ, E-cadherin, Vimentin | EGFR | miR-125a-5p could inhibit EMT of OC cells through affecting TAZ/EGFR pathway. | (Cao et al., 2019) |
| Breast Cancer (BC) | miR-6838-5p | - | CC1937, HCC70, MDA-MB-231, MDA-MB-436, MDA-MB-468, MCF-10A | WNT3A, E-cadherin, N-cadherin, Vimentin | Wnt | miR-6838-5p by targeting WNT3A to inhibit Wnt pathway could reduce cell metastasis and EMT process in BC. | (Liu et al., 2019) |
| BC | miR-199a-3p | - | HCC1806, HCC1937, MDA-MB-231, HMEC-184 | GPER, E-cadherin, N-cadherin, Vimentin, VEGFA, Ang II, CD151 | Hippo | miR-199a-3p could inhibit the EMT process and the Hippo signal pathway in BC. | (Huang et al., 2020) |
| BC | miR-23a | 30 pairs of BC and ACTs | MCF-7, T47D, MDA-MB-468, BT-549, MDA-MB-231 | CDH1, TGF-β1, E-cadherin | Wnt/β-catenin | miR-23a silencing suppresses TGF-β1-induced EMT, migration, invasion and metastasis of BC. | (Ma et al., 2017) |
| BC | miR-200c | - | MDA-MB-231 | UTMD, ZEB1 | ROS | UTMD could inhibit EMT of BC via the ROS/miR-200c/ZEB1 axis. | (Shi et al., 2020) |
| BC | miR-93-5p | - | MCF-7, MDA-MB-231, T47D | MKL-1, STAT3, E-cadherin, N-cadherin, Vimentin | JAK/STAT | miR-93-5p could promote EMT of BC cells. | (Xiang et al., 2017) |
| BC | miR-200c | - | MCF-7, MDA‑MB‑231 | ATA, ZEB-1, E cadherin, occludin, N-cadherin, Vimentin | p53 | miR-200c could inhibit EMT in BC cells by targeting ATA. | (Kumar et al., 2019) |
| BC | miR-145 | - | MCF7, MDAMB231, HepG2 | SMAD3, DR5, BRCA2 | - | miR‑145 promotes proliferation and EMT in BC cells. | (Manvati et al., 2019) |
| BC | miR-125b | 20 pairs of BC and ACTs | MDA-MB-468, MDA-MB-231, MCF-7, MCF-10A | APC, β-catenin, cyclin D1, E-cadherin, Vimentin, Snail | Wnt/β-catenin | Downregulation of miR-125b could inhibit migration, invasion and EMT in BC. | (Nie et al., 2019) |
| BC | miR-92b | 51 pairs of BC and ACTs | MCF-10A, BT549, MDA-MB-231 | Gabra3, N-cadherin, E-cadherin, Vimentin | - | miR-92b could inhibit cells EMT by targeting Gabra3 in BC. | (Li et al., 2019d) |
| BC | miR-200b | - | MDA-MB-231, MCF-7 | ZEB1, ZEB2, E-cadherin | Estradiol/ERβ | miR-200b could inhibit the EMT program in MDA-MB-231 cells depending on ER status and signaling in BC. | (Zoi et al., 2020) |
| BC | miR-205 | 40 pairs of BC and ACTs | MDA-MB-231, MDA-MB-453, MDA-MB-468, MCF-7, MCF-10F | HMGB1, E-cadherin, Vimentin | RAGE | Downregulation of miR-205 could promote EMT and invasion in BC by targeting HMGB1/RAGE pathway. | (Wang et al., 2019a) |
| BC | miR‐516a‐3p | 60 pairs of BC and ACTs | HBL‐100, MDA‐MB‐231, MCF‐7, 293T | Pygo2, E‐cadherin, Vimentin | Wnt/β‐catenin | miR‐516a‐3p could inhibit BC cell growth and EMT by hindering the Pygo2/Wnt axis. | (Chi et al., 2019) |
| BC | miR-365-3p | 93 pairs of BC and ACTs | MCF-7, MDA-MB-231,MCF-10A | FOXK1, β-Actin, E-cadherin, N-cadherin, Vimentin, Slug | - | miR-365-3p could inhibit EMT in BC by targeting FOXK1. | (Gao and Tian 2020) |
| BC | miR-524-5p | 20 pairs of BC and ACTs | SK-BR-3, MDA-MB-453 | FSTL1, MMP2, MMP9, E-cadherin, N-cadherin | - | miR-524-5p could inhibit migration, invasion, and EMT in BC cells via influencing FSTL1. | (Jin et al., 2020) |
| BC | miR-200c | - | Hs578T, SUM159PT, SUM159PT, BT549, MDA-MB-453, MDA-MB-231, MCF7 | TDO2, ZEB-1, E-cadherin | - | miR-200c could inhibit EMT in BC cell lines by targeting TDO2. | (Rogers et al., 2019) |
| BC | miR-425 | 60 pairs of BC and ACTs | MDA-MB-231, 578T | TGF-β1, N-cadherin, Vimentin, E-cadherin | SMAD3 | miR-425 suppresses EMT through targeting the TGF-β 1/SMAD3 axis. | (Yingping and Jinglong 2019) |
| BC | miR-124 | 30 pairs of BC and ACTs | MDA-MB-453, MDA-MB-231, BT-549 | E-cadherin, N-cadherin, Vimentin, ZEB2 | - | Overexpression of miR-124 could inhibit the proliferation, metastasis and EMT of BC cells. | (Ji et al., 2019) |
| BC | miR-335 | - | MCF7, MDA-MB-231 | CDH11, E-cadherin, Vimentin | β-catenin | Upregulation of miR-335 could inhibit EMT in metastatic BC by targeting CDH11. | (Chen et al., 2019) |
| BC | miR-486-5p | - | MCF-7, MCF-10A | N-cadherin, Smad2, Snai1, Fibronectin, Vimentin, E-cadherin | - | miR-486-5p blocks proliferation, migration, invasion, and EMT by regulating SMAD2 in BC. | (K et al., 2017) |
| BC | miR-27a | 20 pairs of BC and ACTs | MDA-MB-231, SKBR3, MCF-12A | FBXW7, ZEB1, E-cadherin, N-cadherin, Vimentin | - | miR-27a could promote EMT of BC. | (Jiang et al., 2018) |
| Renal Cell Carcinoma (RCC) | miR-452-5p | 20 pairs of RCC and ACTs | OSRC-2, SW839, A498, SN12-PM6 | SMAD4/7, N-cadherin, E-cadherin, Vimentin | p65 | miR-452-5p could promote EMT in RCC by targeting SMAD4/p65 axis. | (Zhai et al., 2018) |
| Bladder Cancer (BLC) | miR-221 | 117 pairs of BCL and ACTs | CCC-HB-2, UMUC3, SVHUC1, T24, J82, 5637 | circMTO1, E-cadherin, N-cadherin | - | miR-221 induction by CircMTO1 could inhibit EMT in BLC. | (Li et al., 2019c) |
| BLC | miR-30a | 15 pairs of BCL and ACTs | HTB-1, T24 | BAY11-7082, Snail, Vimentin, VEGF, E-cadherin | NF-κB | miR-30a could inhibit EMT in BLC cells. | (Zhang et al., 2019c) |
| BLC | miR-19a | 59 pairs of BCL and ACTs | SV-HUC-1, J82, T24 | RhoB, N-cadherin, E-cadherin, Vimentin | - | miR-19a promotes invasion and EMT of BLC cells by targeting RhoB. | (Li et al., 2019e) |
| BLC | miR-381-3p | 9 pairs of BCL and ACTs | T24, UM-UC3, 5637, SV-HUC-1, 293T | CDK6, CCNA2, MET, SNAIL, N-cadherin, E-cadherin, Vimentin | ROCK/Akt/β-catenin, MET | miR-381-3p down-regulates MET and CCNA2 induced EMT progression. | (Li et al., 2019a) |
| BLC | miR-506 | 40 pairs of BCL and ACTs | T24, J82, UM-UC-3, SV-HUC-1 | RWDD4, N-cadherin, E-cadherin, Vimentin | - | miR-506 could inhibit cell proliferation, invasion, migration, and EMT by targeting RWDD4 in BLC. | (Hou 2019) |
| Pancreatic Cancer (PaC) | miR-202-5p | - | MCF-7, PAN02, KPC, PSCs | DZNep, TGFBR1, TGFBR2, N-cadherin, E-cadherin, Vimentin | TGF-β | miR-202 could suppress TGF-β signaling and EMT phenotypic characteristics of BLC cells. | (Mody et al., 2017) |
| PaC | miR-135b-5p | 80 pairs of PaC and ACTs | HPDE6c7, PANC-1, AsPC-1, SW1990, BxPC-3 | NR3C2 E-cadherin, Vimentin, β-catenin | - | miR-135b-5p could promote migratory potential, invasion, and EMT through targeting NR3C2. | (Zhang et al., 2017d) |
| PaC | miR-203a-3p | 20 pairs of PaC and ACTs | PANC-1, AsPC-1, Capan-1, SW1990, HPC-Y5 | SLUG, E-Cadherin, N-Cadherin | - | miR-203a-3p could inhibit PaC cell proliferation, EMT, and apoptosis by modulating SLUG. | (An and Zheng 2020) |
| PaC | miR-3656 | 157 pairs of PaC and ACTs | HPDE6-C7, HPNE, Capan-2, HPAC, SW1990, PANC-1, CFPAC-1, BXPC-3, ASPC-1, PATU-8988 | RHOF, E-cadherin, N-cadherin, Vimentin | - | Downregulation of miR-3656 could promote EMT process by modulating the RHOF/EMT axis. | (Yang et al., 2017b) |
| Gastric Cancer (GC) | miR-375 | - | MGC-803, GES-1, SGC-7901, HGC-27, BGC-823 | YWHAZ, E-cadherin, N-cadherin, Vimentin | Wnt/β-catenin | Downregulation of miR-375 via the Wnt/β-catenin pathway could promote EMT in GC cells. | (Guo et al., 2019) |
| GC | miR-203 | 10 pairs of GC and ACTs | AGS, BGC-823, MKN45, SGC7901, GES-1 | Annexin A4, ZEB1, E-cadherin, N-cadherin, Vimentin, Twist1, Slug | - | miR-203 could inhibit the invasion and EMT through modulating Annexin A4. | (Li et al., 2019b) |
| GC | miR-302b | - | SGC-7901, AGS | EphA2, N-cadherin, E-cadherin | Wnt/β-catenin | miR-302b could promote EMT by targeting EphA2 in GC cells. | (Huang et al., 2017) |
| GC | miR-95 | 63 pairs of GC and ACTs | CTC-141, MKN45, GES-1 | Slug, E-cadherin, N-cadherin, Vimentin | - | Downregulation of miR-95 could promote EMT via regulation of Slug in GC. | (Zhang et al., 2019d) |
| GC | miR-125a-5p | 35 pairs of GC and ACTs | BGC823, MKN28, AGS, HGC-27, SGC7901, GES-1 | FOXS1, N-cadherin, Vimentin, E-cadherin | Wnt/β-catenin, Hh | miR-125a-5p could inhibit EMT and cell proliferation in GC by targeting FOXS1. | (Wang et al., 2019b) |
| GC | miR-646 | 74 pairs of GC and ACTs | GES-1 | FOXK1, TGF-β1, β-catenin, E-cadherin, Vimentin | Akt/mTOR | miR-646 suppresses EMT by affecting FOXK1 expression in GC. | (Zhang et al., 2017c) |
| GC | miR-218 | - | SGC7901, BGC823 | WASF3, E-cadherin, N-cadherin, Vimentin | - | miR-218 could inhibit proliferation, migration, and EMT of GC cells by affecting WASF3. | (Wang et al., 2017b) |
| GC | miR-200c | - | SGC7901, BGC803, MKN28, NCI-N87 | ZEB1, ZEB2, E-cadherin, N-cadherin | TGF-β | miR-200c could inhibit TGF-β-induced-EMT by targeting ZEB1 and ZEB2 in GC. | (Zhou et al., 2018) |
| GC | miR-204 | 60 pairs of GC and ACTs | GC cell lines | Snai1 | - | miR-204 modulates EMT through influencing snai1 expression. | (Liu et al., 2016) |
| CC | miR-31-3p | 52 pairs of CC and ACTs | HeLa, CaSki, SiHa, C33a | Sema4C, E-cadherin, Vimentin, Snail | - | Overexpression of miR-31-3p could inhibit EMT in CC. | (Jing et al., 2019) |
| CC | miR-211 | 8 CC tissues and 3 normal cervix tissues | HeLa, SiHa, | MUC4, E-cadherin, N-cadherin, Vimentin | - | miR-211 inhibits invasiveness and EMT of CC cells via targeting MUC4. | (Xu et al., 2017) |
| CC | miR-338-3p | 45 pairs of CC and ACTs | HeLa, CaSki, SiHa, C-33A, C-4I, SW756, End1/E6E7 | HIF-1α, HIPK3 | - | miR-338-3p could promote CC cell growth, migration, and EMT by targeting HIF-1α. | (Qian et al., 2020) |
| Colorectal Cancer (CRC) | miR‐330 | 300 pairs of CRC and ACTs | HCT116, SW480 | HMGA2, Smad3, E‐cadherin | TGF-β | miR-330 could inhibit EMT and prompts apoptosis by decreasing HMGA2 in CRC. | (Mansoori et al., 2020) |
| CRC | miR-145-5p | 30 pairs of CRC and ACTs | LoVo, HT29, SW480, SW620, NCM460 | CDCA3, E-cadherin, N-cadherin | - | miR-145-5p could inhibit EMT of CRC by targeting CDCA3. | (Chen et al., 2020) |
| CRC | miR-383-5p | 35 pairs of CRC and ACTs | LoVo, HCT116, NCM460 | SGK1, N-cadherin, E-cadherin | - | miR-383-5p could inhibit cell proliferation, metastasis, and EMT in CRC cells by influencing expression of SGK1. | (Chong et al., 2019) |
| CRC | miR-3622a-3p | 80 pairs of CRC and ACTs | HCT116, SW480, HT-29, LoVo, DLD-1, NCM460 | SALL4, E-cadherin, N-cadherin, Vimentin, Snail | Wnt/β-catenin | miR-3622a-3p by targeting SALL4 via the Wnt/β-catenin pathway could inhibit EMT in CRC. | (Chang et al., 2020) |
| CRC | miR-205 | 47 pairs of CRC and ACTs | HCT116, HT29, HCT8, LS174T, SW480, 293T | MDM4, E-cadherin, N-cadherin, Vimentin, MMP, MMP9 | - | miR-205 could inhibit cell migration, invasion and EMT of CRC by targeting MDM4. | (Fan and Wang 2020) |
| CRC | miR-200b | 50 pairs of CRC and ACTs | LS174T, SW480 | HIF-1α, Ascl2, E-cadherin, N-cadherin | - | Overexpression of miR-200b by targeting HIF-1α could inhibit EMT in CRC. | (Shang et al., 2017) |
| CRC | miR-147 | - | HCT116, SW480, HCT-8, LS174T, CCD 841 | TGF-β1, E-cadherin, Vimentin | Wnt/β-catenin | miR-147 could inhibit EMT in CRC Cells. | (Ning et al., 2019) |
| CRC | miR-138 | - | SW480, SW1116, LOVO, HCT116, FHC | PODXL, N-cadherin, Vimentin | Wnt/β-catenin | miR-138 by targeting PODXL could promote CRC cells proliferation, migration, invasion and EMT. | (Xu et al., 2018) |
| CRC | miR-519d | 20 pairs of CRC and ACTs | THC-8307, HCT116 | DRIP1, E-cadherin, Vimentin | - | miR-519d by targeting DRIP1 could inhibit EMT in CRC. | (Yang et al., 2017c) |
| CRC | miR-495 | 32 pairs of CRC and ACTs | SW620, SW480, HT29, HCT116, FHC | Annexin A3, Vimentin, E-cadherin, β-catenin, Fibronectin | p53 | miR-495 could inhibit the invasion and EMT by targeting Annexin A3/p53 axis. | (Bai et al., 2017) |
| CRC | miR-598 | 4 pairs of CRC and ACTs | HT-29, HCT116, SW620, NCM-460 | JAG1, E-cadherin, N-cadherin, Vimentin | Notch2 | Downregulation of miR-598 could inhibit EMT in CRC via Notch2 pathway. | (Chen et al., 2017) |
| CRC | miR-200 | 62 pairs of CRC and ACTs | Caco-2, LS174T, Lovo, HT-29, HCT116, SW480, SW620 | Nanog, E-cadherin, N-cadherin | - | Downregulation of miR-200 by Nanog in CRC cells could induce EMT. | (Pan et al., 2017) |
| CRC | miR-30a | 80 pairs of CRC and ACTs | Caco-2, LOVO, SW480, DLD-1 | TM4SF1, E-cadherin, β-catenin | - | miR-30a could inhibit EMT in CRC by targeting TM4SF1. | (Park et al., 2017) |
| CRC | miR-429 | Stage II and stage III samples and ACTs | SW-480, SW-620, HT-29 | ONECUT2 | - | miR-429 suppresses growth and invasiveness and modulates EMT-associated markers through influencing Onecut2 expression. | (Sun et al., 2014) |
| Endometrial cancer (EC) | miR-214-3p | 22 pairs of EC and ACTs | HEC-1-A, HEC-1-B, RL95-2, HEK293, hEECs | TWIST1, Fibronectin, E-cadherin, N-cadherin | - | mir-214-3p could inhibit EMT and metastasis of EC cells by targeting TWIST1. | (Fang et al., 2019) |
| EC | miR-326 | 56 pairs of CRC and ACTs | HEC-1B, RL95-2, AN3CA, NEEC | TWIST1, E-cadherin, N-cadherin, Vimentin, Fibronectin | - | miR-326 could inhibit EC cell migration and invasion by suppressing EMT. | (Liu et al., 2017) |
| Gallbladder Cancer | miR-143-5p | 36 pairs of GBC and ACTs | GBC-SD, SGC-996, NOZ | Twist1, E-cadherin, β-catenin, Vimentin | HIF-1α/EMT | miR-143-5p could reduce EMT in GBC by targeting HIF-1α. | (He et al., 2017) |
| Glioblastoma (GBM) | miR-96 | - | U251 | AE-1, E-cadherin, Vimentin | - | miR-96 could inhibit the EMT process by down regulating AEG-1 in GBM. | (Feng et al., 2018) |
| Esophageal Squamous Cell Carcinoma | miR-140-5p | 68 pairs of ESCC and ACTs | eca109, EC9706, TE1, Kyse-30, Kyse-70, HEEC | ZEB1, E-cadherin, N-cadherin, β-catenin | - | Overexpressed miR-140-5p could inhibit EMT in ESCC by targeting ZEB1. | (Zhang et al., 2017a) |
| Hepatocellular carcinoma | miR-187-3p | 120 pairs of cancerous tissues and ACTs | MHCC97H, SMMC7721, HepG2, Huh7, Hep3B | S100A4 | - | miR-187-3p suppresses EMT. | (Dou et al., 2016) |

**References**

Amaar, Y. G. and Reeves, M. E. (2019). RASSF1C regulates miR-33a and EMT marker gene expression in lung cancer cells. Oncotarget 10 123-132.

An, N. and Zheng, B. (2020). MiR-203a-3p Inhibits Pancreatic Cancer Cell Proliferation, EMT, and Apoptosis by Regulating SLUG. Technol Cancer Res Treat 19 1533033819898729.

Bai, Z., Wang, J., Wang, T., Li, Y., Zhao, X., Wu, G., et al. (2017). The MiR-495/Annexin A3/P53 Axis Inhibits the Invasion and EMT of Colorectal Cancer Cells. Cell Physiol Biochem 44 1882-1895.

Basu, S., Chaudhary, A., Chowdhury, P., Karmakar, D., Basu, K., Karmakar, D., et al. (2020). Evaluating the role of hsa-miR-200c in reversing the epithelial to mesenchymal transition in prostate cancer. Gene 730 144264.

Cao, Y., Shen, T., Zhang, C., Zhang, Q. H. and Zhang, Z. Q. (2019). MiR-125a-5p inhibits EMT of ovarian cancer cells by regulating TAZ/EGFR signaling pathway. Eur Rev Med Pharmacol Sci 23 8249-8256.

Chang, S., Sun, G., Zhang, D. and al., e. (2020). MiR-3622a-3p acts as a tumor suppressor in colorectal cancer by reducing stemness features and EMT through targeting spalt-like transcription factor 4. Research Square.

Chang, Z. (2019). Downregulation of SOX2 may be targeted by miR-590-5p and inhibits epithelial-to-mesenchymal transition in non-small-cell lung cancer. Exp Ther Med 18 1189-1195.

Chen, J., Zhang, H., Chen, Y., Qiao, G., Jiang, W., Ni, P., et al. (2017). miR-598 inhibits metastasis in colorectal cancer by suppressing JAG1/Notch2 pathway stimulating EMT. Exp Cell Res 352 104-112.

Chen, J. H., Huang, W. C., Bamodu, O. A., Chang, P. M., Chao, T. Y. and Huang, T. H. (2019). Monospecific antibody targeting of CDH11 inhibits epithelial-to-mesenchymal transition and represses cancer stem cell-like phenotype by up-regulating miR-335 in metastatic breast cancer, in vitro and in vivo. BMC Cancer 19 634.

Chen, Q., Zhou, L., Ye, X., Tao, M. and Wu, J. (2020). miR-145-5p suppresses proliferation, metastasis and EMT of colorectal cancer by targeting CDCA3. Pathol Res Pract 216 152872.

Chi, Y., Wang, F., Zhang, T., Xu, H., Zhang, Y., Shan, Z., et al. (2019). miR-516a-3p inhibits breast cancer cell growth and EMT by blocking the Pygo2/Wnt signalling pathway. J Cell Mol Med 23 6295-6307.

Chong, L., Lingling, S. and Jiaying, S. (2019). Circular RNA hsa_circ_0000467 modulates SGK1 to facilitate cell migration, metastasis, and EMT while repressing apoptosis in colorectal cancer by sponging miR-383-5p. RSC Adv 9 39294-39303.

Dai, L., Chen, F., Zheng, Y., Zhang, D., Qian, B., Ji, H., et al. (2019a). miR-21 regulates growth and EMT in lung cancer cells via PTEN/Akt/GSK3beta signaling. Front Biosci (Landmark Ed) 24 1426-1439.

Dai, Y., Wu, Z., Lang, C., Zhang, X., He, S., Yang, Q., et al. (2019b). Copy number gain of ZEB1 mediates a double-negative feedback loop with miR-33a-5p that regulates EMT and bone metastasis of prostate cancer dependent on TGF-beta signaling. Theranostics 9 6063-6079.

Dong, S., Wang, R., Wang, H., Ding, Q., Zhou, X., Wang, J., et al. (2019). HOXD-AS1 promotes the epithelial to mesenchymal transition of ovarian cancer cells by regulating miR-186-5p and PIK3R3. J Exp Clin Cancer Res 38 110.

Dou, C., Liu, Z., Xu, M., Jia, Y., Wang, Y., Li, Q., et al. (2016). miR-187-3p inhibits the metastasis and epithelial-mesenchymal transition of hepatocellular carcinoma by targeting S100A4. Cancer Lett 381 380-390.

Du, W., Sun, L., Liu, T., Zhu, J., Zeng, Y., Zhang, Y., et al. (2020). The miR6253p/AXL axis induces nonT790M acquired resistance to EGFRTKI via activation of the TGFbeta/Smad pathway and EMT in EGFRmutant nonsmall cell lung cancer. Oncol Rep.

Fan, Y. and Wang, K. (2020). miR‑205 suppresses cell migration, invasion and EMT of colon cancer by targeting mouse double minute 4. Molecular Medicine Reports.

Fang, Y. Y., Tan, M. R., Zhou, J., Liang, L., Liu, X. Y., Zhao, K. and Bao, E. C. (2019). miR-214-3p inhibits epithelial-to-mesenchymal transition and metastasis of endometrial cancer cells by targeting TWIST1. Onco Targets Ther 12 9449-9458.

Feng, S., Yao, J., Zhang, Z., Zhang, Y., Zhang, Z., Liu, J., et al. (2018). miR96 inhibits EMT by targeting AEG1 in glioblastoma cancer cells. Mol Med Rep 17 2964-2972.

Gao, F. and Tian, J. (2020). FOXK1, Regulated by miR-365-3p, Promotes Cell Growth and EMT Indicates Unfavorable Prognosis in Breast Cancer. Onco Targets Ther 13 623-634.

Guo, F., Gao, Y., Sui, G., Jiao, D., Sun, L., Fu, Q. and Jin, C. (2019). miR-375-3p/YWHAZ/beta-catenin axis regulates migration, invasion, EMT in gastric cancer cells. Clin Exp Pharmacol Physiol 46 144-152.

He, M., Zhan, M., Chen, W., Xu, S., Long, M., Shen, H., et al. (2017). MiR-143-5p Deficiency Triggers EMT and Metastasis by Targeting HIF-1α in Gallbladder Cancer. Cell Physiol Biochem 42 2078-2092.

Hou, Y. (2019). MiR-506 inhibits cell proliferation, invasion, migration and epithelial-to-mesenchymal transition through targeting RWDD4 in human bladder cancer. Oncol Lett 17 73-78.

Hu, W., Yan, F., Ru, Y., Xia, M., Yan, G., Zhang, M., et al. (2020). MIIP inhibits EMT and cell invasion in prostate cancer through miR-181a/b-5p-KLF17 axis. Am J Cancer Res 10 630-647.

Huang, J., He, Y., McLeod, H. L., Xie, Y., Xiao, D., Hu, H., et al. (2017). miR-302b inhibits tumorigenesis by targeting EphA2 via Wnt/ beta-catenin/EMT signaling cascade in gastric cancer. BMC Cancer 17 886.

Huang, R., Li, J., Pan, F., Zhang, B. and Yao, Y. (2020). The activation of GPER inhibits cells proliferation, invasion and EMT of triple-negative breast cancer via CD151/miR-199a-3p bio-axis. Am J Transl Res 12 32-44.

Ji, H., Sang, M., Liu, F., Ai, N. and Geng, C. (2019). miR-124 regulates EMT based on ZEB2 target to inhibit invasion and metastasis in triple-negative breast cancer. Pathol Res Pract 215 697-704.

Jiang, G., Shi, W., Fang, H. and Zhang, X. (2018). miR27a promotes human breast cancer cell migration by inducing EMT in a FBXW7dependent manner. Mol Med Rep 18 5417-5426.

Jiao, D., Chen, J., Li, Y., Tang, X., Wang, J., Xu, W., et al. (2018). miR-1-3p and miR-206 sensitizes HGF-induced gefitinib-resistant human lung cancer cells through inhibition of c-Met signalling and EMT. J Cell Mol Med 22 3526-3536.

Jin, T., Zhang, Y. and Zhang, T. (2020). MiR-524-5p Suppresses Migration, Invasion, and EMT Progression in Breast Cancer Cells Through Targeting FSTL1. Cancer Biother Radiopharm.

Jing, L., Bo, W., Yourong, F., Tian, W., Shixuan, W. and Mingfu, W. (2019). Sema4C mediates EMT inducing chemotherapeutic resistance of miR-31-3p in cervical cancer cells. Sci Rep 9 17727.

K, T., G, H. and Q, F. (2017). MiR-486-5p prevents migration, invasion and EMT by regulating smad2 in breast cancer. Int J Clin Exp Med 10 8942-8949.

Kumar, K. J. S., Vani, M. G., Hsieh, H. W., Lin, C. C. and Wang, S. Y. (2019). Antcin-A Modulates Epithelial-to-Mesenchymal Transition and Inhibits Migratory and Invasive Potentials of Human Breast Cancer Cells via p53-Mediated miR-200c Activation. Planta Med 85 755-765.

Li, J., Ying, Y., Xie, H., Jin, K., Yan, H., Wang, S., et al. (2019a). Dual regulatory role of CCNA2 in modulating CDK6 and MET-mediated cell-cycle pathway and EMT progression is blocked by miR-381-3p in bladder cancer. Faseb j 33 1374-1388.

Li, J., Zhang, B., Cui, J., Liang, Z. and Liu, K. (2019b). miR-203 Inhibits the Invasion and EMT of Gastric Cancer Cells by Directly Targeting Annexin A4. Oncol Res 27 789-799.

Li, X., Lin, S., Mo, Z., Jiang, J., Tang, H., Wu, C. and Song, J. (2020). CircRNA_100395 inhibits cell proliferation and metastasis in ovarian cancer via regulating miR-1228/p53/epithelial-mesenchymal transition (EMT) axis. J Cancer 11 599-609.

Li, Y., Wan, B., Liu, L., Zhou, L. and Zeng, Q. (2019c). Circular RNA circMTO1 suppresses bladder cancer metastasis by sponging miR-221 and inhibiting epithelial-to-mesenchymal transition. Biochem Biophys Res Commun 508 991-996.

Li, Y. Y., Zheng, X. H., Deng, A. P., Wang, Y., Liu, J., Zhou, Q., et al. (2019d). MiR-92b inhibited cells EMT by targeting Gabra3 and predicted prognosis of triple negative breast cancer patients. Eur Rev Med Pharmacol Sci 23 10433-10442.

Li, Z., Li, Y. and Wang, Y. (2019e). miR-19a promotes invasion and epithelial to mesenchymal transition of bladder cancer cells by targeting RhoB. J buon 24 797-804.

Liang, H., Yu, M., Yang, R., Zhang, L., Zhang, L., Zhu, D., et al. (2020). A PTAL-miR-101-FN1 Axis Promotes EMT and Invasion-Metastasis in Serous Ovarian Cancer. Mol Ther Oncolytics 16 53-62.

Lin, X., Lin, B. W., Chen, X. L., Zhang, B. L., Xiao, X. J., Shi, J. S., et al. (2017). PAI-1/PIAS3/Stat3/miR-34a forms a positive feedback loop to promote EMT-mediated metastasis through Stat3 signaling in Non-small cell lung cancer. Biochem Biophys Res Commun 493 1464-1470.

Liu, G., Wang, P. and Zhang, H. (2019). MiR-6838-5p suppresses cell metastasis and the EMT process in triple-negative breast cancer by targeting WNT3A to inhibit the Wnt pathway. J Gene Med 21 e3129.

Liu, W., Zhang, B., Xu, N., Wang, M. J. and Liu, Q. (2017). miR-326 regulates EMT and metastasis of endometrial cancer through targeting TWIST1. Eur Rev Med Pharmacol Sci 21 3787-3793.

Liu, Z., Long, J., Du, R., Ge, C., Guo, K. and Xu, Y. (2016). miR-204 regulates the EMT by targeting snai1 to suppress the invasion and migration of gastric cancer. Tumour Biol 37 8327-8335.

Ma, F., Li, W., Liu, C., Li, W., Yu, H., Lei, B., et al. (2017). MiR-23a promotes TGF-beta1-induced EMT and tumor metastasis in breast cancer cells by directly targeting CDH1 and activating Wnt/beta-catenin signaling. Oncotarget 8 69538-69550.

Mansoori, B., Mohammadi, A., Naghizadeh, S., Gjerstorff, M., Shanehbandi, D., Shirjang, S., et al. (2020). miR-330 suppresses EMT and induces apoptosis by downregulating HMGA2 in human colorectal cancer. J Cell Physiol 235 920-931.

Manvati, S., Mangalhara, K. C., Kalaiarasan, P., Chopra, R., Agarwal, G., Kumar, R., et al. (2019). miR-145 supports cancer cell survival and shows association with DDR genes, methylation pattern, and epithelial to mesenchymal transition. Cancer Cell Int 19 230.

Mody, H. R., Hung, S. W., Pathak, R. K., Griffin, J., Cruz-Monserrate, Z. and Govindarajan, R. (2017). miR-202 Diminishes TGFbeta Receptors and Attenuates TGFbeta1-Induced EMT in Pancreatic Cancer. Mol Cancer Res 15 1029-1039.

Nie, J., Jiang, H. C., Zhou, Y. C., Jiang, B., He, W. J., Wang, Y. F. and Dong, J. (2019). MiR-125b regulates the proliferation and metastasis of triple negative breast cancer cells via the Wnt/beta-catenin pathway and EMT. Biosci Biotechnol Biochem 83 1062-1071.

Ning, X., Wang, C., Zhang, M. and Wang, K. (2019). Ectopic Expression of miR-147 Inhibits Stem Cell Marker and Epithelial-Mesenchymal Transition (EMT)-Related Protein Expression in Colon Cancer Cells. Oncol Res 27 399-406.

Pan, Q., Meng, L., Ye, J., Wei, X., Shang, Y., Tian, Y., et al. (2017). Transcriptional repression of miR-200 family members by Nanog in colon cancer cells induces epithelial-mesenchymal transition (EMT). Cancer Lett 392 26-38.

Park, Y. R., Kim, S. L., Lee, M. R., Seo, S. Y., Lee, J. H., Kim, S. H., et al. (2017). MicroRNA-30a-5p (miR-30a) regulates cell motility and EMT by directly targeting oncogenic TM4SF1 in colorectal cancer. J Cancer Res Clin Oncol 143 1915-1927.

Qi, J. C., Yang, Z., Zhang, Y. P., Lu, B. S., Yin, Y. W., Liu, K. L., et al. (2019). miR-20b-5p, TGFBR2, and E2F1 Form a Regulatory Loop to Participate in Epithelial to Mesenchymal Transition in Prostate Cancer. Front Oncol 9 1535.

Qian, W., Huang, T. and Feng, W. (2020). Circular RNA HIPK3 Promotes EMT of Cervical Cancer Through Sponging miR-338-3p to Up-Regulate HIF-1alpha. Cancer Manag Res 12 177-187.

Ren, D., Yang, Q., Dai, Y., Guo, W., Du, H., Song, L. and Peng, X. (2017). Oncogenic miR-210-3p promotes prostate cancer cell EMT and bone metastasis via NF-kappaB signaling pathway. Mol Cancer 16 117.

Rogers, T. J., Christenson, J. L., Greene, L. I., O'Neill, K. I., Williams, M. M., Gordon, M. A., et al. (2019). Reversal of Triple-Negative Breast Cancer EMT by miR-200c Decreases Tryptophan Catabolism and a Program of Immunosuppression. Mol Cancer Res 17 30-41.

Sato, H., Shien, K., Tomida, S., Okayasu, K., Suzawa, K., Hashida, S., et al. (2017). Targeting the miR-200c/LIN28B axis in acquired EGFR-TKI resistance non-small cell lung cancer cells harboring EMT features. Sci Rep 7 40847.

Shang, Y., Chen, H., Ye, J., Wei, X., Liu, S. and Wang, R. (2017). HIF-1alpha/Ascl2/miR-200b regulatory feedback circuit modulated the epithelial-mesenchymal transition (EMT) in colorectal cancer cells. Exp Cell Res 360 243-256.

Shi, D., Guo, L., Sun, X., Shang, M., Meng, D., Zhou, X., et al. (2020). UTMD inhibit EMT of breast cancer through the ROS/miR-200c/ZEB1 axis. Sci Rep 10 6657.

Shi, L., Wang, Y., Lu, Z., Zhang, H., Zhuang, N., Wang, B., et al. (2017). miR-127 promotes EMT and stem-like traits in lung cancer through a feed-forward regulatory loop. Oncogene 36 1631-1643.

Su, C., Cheng, X., Li, Y., Han, Y., Song, X., Yu, D., et al. (2018). MiR-21 improves invasion and migration of drug-resistant lung adenocarcinoma cancer cell and transformation of EMT through targeting HBP1. Cancer Med 7 2485-2503.

Sun, Y., Shen, S., Liu, X., Tang, H., Wang, Z., Yu, Z., et al. (2014). MiR-429 inhibits cells growth and invasion and regulates EMT-related marker genes by targeting Onecut2 in colorectal carcinoma. Mol Cell Biochem 390 19-30.

Tao, L., Shu-Ling, W., Jing-Bo, H., Ying, Z., Rong, H., Xiang-Qun, L., et al. (2020). MiR-451a attenuates doxorubicin resistance in lung cancer via suppressing epithelialmesenchymal transition (EMT) through targeting c-Myc. Biomed Pharmacother 125 109962.

Wang, D. X., Zou, Y. J., Zhuang, X. B., Chen, S. X., Lin, Y., Li, W. L., et al. (2017a). Sulforaphane suppresses EMT and metastasis in human lung cancer through miR-616-5p-mediated GSK3β/β-catenin signaling pathways. Acta Pharmacol Sin 38 241-251.

Wang, G., Fu, Y., Liu, G., Ye, Y. and Zhang, X. (2017b). miR-218 Inhibits Proliferation, Migration, and EMT of Gastric Cancer Cells by Targeting WASF3. Oncol Res 25 355-364.

Wang, H. Y., Liu, Y. N., Wu, S. G., Hsu, C. L., Chang, T. H., Tsai, M. F., et al. (2020a). MiR-200c-3p suppression is associated with development of acquired resistance to epidermal growth factor receptor (EGFR) tyrosine kinase inhibitors in EGFR mutant non-small cell lung cancer via a mediating epithelial-to-mesenchymal transition (EMT) process. Cancer Biomark 28 351-363.

Wang, L., Kang, F. B., Wang, J., Yang, C. and He, D. W. (2019a). Downregulation of miR-205 contributes to epithelial-mesenchymal transition and invasion in triple-negative breast cancer by targeting HMGB1-RAGE signaling pathway. Anticancer Drugs 30 225-232.

Wang, S., Ran, L., Zhang, W., Leng, X., Wang, K., Liu, G., et al. (2019b). FOXS1 is regulated by GLI1 and miR-125a-5p and promotes cell proliferation and EMT in gastric cancer. Sci Rep 9 5281.

Wang, S., Wang, X., Li, J., Meng, S., Liang, Z., Xu, X., et al. (2017c). c-Met, CREB1 and EGFR are involved in miR-493-5p inhibition of EMT via AKT/GSK-3β/Snail signaling in prostate cancer. Oncotarget 8 82303-82313.

Wang, S., Wu, Y., Yang, S., Liu, X., Lu, Y., Liu, F., et al. (2020b). miR-874 directly targets AQP3 to inhibit cell proliferation, mobility and EMT in non-small cell lung cancer. Thorac Cancer.

Xiang, Y., Liao, X. H., Yu, C. X., Yao, A., Qin, H., Li, J. P., et al. (2017). MiR-93-5p inhibits the EMT of breast cancer cells via targeting MKL-1 and STAT3. Exp Cell Res 357 135-144.

Xu, D., Liu, S., Zhang, L. and Song, L. (2017). MiR-211 inhibits invasion and epithelial-to-mesenchymal transition (EMT) of cervical cancer cells via targeting MUC4. Biochem Biophys Res Commun 485 556-562.

Xu, Y., Pan, Z. G., Shu, L. and Li, Q. J. (2018). Podocalyxin-like, targeted by miR-138, promotes colorectal cancer cell proliferation, migration, invasion and EMT. Eur Rev Med Pharmacol Sci 22 8664-8674.

Xue, Y. B., Ding, M. Q., Xue, L. and Luo, J. H. (2019). CircAGFG1 sponges miR-203 to promote EMT and metastasis of non-small-cell lung cancer by upregulating ZNF281 expression. Thorac Cancer 10 1692-1701.

Yang, D., Ma, M., Zhou, W., Yang, B. and Xiao, C. (2017a). Inhibition of miR-32 activity promoted EMT induced by PM2.5 exposure through the modulation of the Smad1-mediated signaling pathways in lung cancer cells. Chemosphere 184 289-298.

Yang, R. M., Zhan, M., Xu, S. W., Long, M. M., Yang, L. H., Chen, W., et al. (2017b). miR-3656 expression enhances the chemosensitivity of pancreatic cancer to gemcitabine through modulation of the RHOF/EMT axis. Cell Death Dis 8 e3129.

Yang, X., Hu, Y., Liu, Y., Liu, W., Zhao, X., Liu, M. and Tang, H. (2017c). C14orf28 downregulated by miR-519d contributes to oncogenicity and regulates apoptosis and EMT in colorectal cancer. Mol Cell Biochem 434 197-208.

Yingping, L. and Jinglong, C. (2019). miR-425 suppresses EMT and the development of TNBC (triple-negative breast cancer) by targeting the TGF-b1/SMAD 3 signaling pathway. RSC Adv 9 151-165.

Zhai, W., Li, S., Zhang, J., Chen, Y., Ma, J., Kong, W., et al. (2018). Sunitinib-suppressed miR-452-5p facilitates renal cancer cell invasion and metastasis through modulating SMAD4/SMAD7 signals. Mol Cancer 17 157.

Zhang, K., Chen, J., Song, H. and Chen, L.-B. (2017a). SNHG16/miR-140-5p axis promotes esophagus cancer cell proliferation, migration and EMT formation through regulating ZEB1. Oncotarget 9 1028-1040.

Zhang, K., Li, X. Y., Wang, Z. M., Han, Z. F. and Zhao, Y. H. (2017b). MiR-22 inhibits lung cancer cell EMT and invasion through targeting Snail. Eur Rev Med Pharmacol Sci 21 3598-3604.

Zhang, L., Liu, X. L., Yuan, Z., Cui, J. and Zhang, H. (2019a). MiR-99a suppressed cell proliferation and invasion by directly targeting HOXA1 through regulation of the AKT/mTOR signaling pathway and EMT in ovarian cancer. Eur Rev Med Pharmacol Sci 23 4663-4672.

Zhang, L. Y., Chen, Y., Jia, J., Zhu, X., He, Y. and Wu, L. M. (2019b). MiR-27a promotes EMT in ovarian cancer through active Wnt/-catenin signalling by targeting FOXO1. Cancer Biomark 24 31-42.

Zhang, P., Tang, W. M., Zhang, H., Li, Y. Q., Peng, Y., Wang, J., et al. (2017c). MiR-646 inhibited cell proliferation and EMT-induced metastasis by targeting FOXK1 in gastric cancer. Br J Cancer 117 525-534.

Zhang, Q., Mao, Z. and Sun, J. (2019c). NF-κB inhibitor, BAY11-7082, suppresses M2 tumor-associated macrophage induced EMT potential via miR-30a/NF-κB/Snail signaling in bladder cancer cells. Gene 710 91-97.

Zhang, W., Sun, J., Chen, J., Xu, C. and Zhang, L. (2019d). Downregulation of miR-95 in gastric cancer promotes EMT via regulation of Slug, thereby promoting migration and invasion. Oncol Rep 41 1395-1403.

Zhang, Z., Che, X., Yang, N., Bai, Z., Wu, Y., Zhao, L. and Pei, H. (2017d). miR-135b-5p Promotes migration, invasion and EMT of pancreatic cancer cells by targeting NR3C2. Biomed Pharmacother 96 1341-1348.

Zhang, Z., Yang, Y. and Zhang, X. (2017e). MiR-770 inhibits tumorigenesis and EMT by targeting JMJD6 and regulating WNT/beta-catenin pathway in non-small cell lung cancer. Life Sci 188 163-171.

Zhao, Z., Zhao, S., Luo, L., Xiang, Q., Zhu, Z., Wang, J., et al. (2021). miR-199b-5p-DDR1-ERK signalling axis suppresses prostate cancer metastasis via inhibiting epithelial-mesenchymal transition. Br J Cancer 124 982-994.

Zheng, F., Li, J., Ma, C., Tang, X., Tang, Q., Wu, J., et al. (2020). Novel regulation of miR-34a-5p and HOTAIR by the combination of berberine and gefitinib leading to inhibition of EMT in human lung cancer. J Cell Mol Med 24 5578-5592.

Zhou, X., Men, X., Zhao, R., Han, J., Fan, Z., Wang, Y., et al. (2018). miR-200c inhibits TGF-beta-induced-EMT to restore trastuzumab sensitivity by targeting ZEB1 and ZEB2 in gastric cancer. Cancer Gene Ther 25 68-76.

Zoi, P., Marco, F., Christoph, R., Martin, G. and Nikos K, K. (2020). miR-200b restrains EMT and aggressiveness and regulates matrix composition depending on ER status and signaling in mammary cancer. Matrix Biology Plus.
