# Supplementary material for: The Impact of Non-coding RNAs in the Epithelial to Mesenchymal Transition
Source: Front Mol Biosci. 2021 Mar 26;8:665199. doi: 10.3389/fmolb.2021.665199 (PMC8033041; doi:10.3389/fmolb.2021.665199)
Supplement: Supplementary file 2 [file Table_2.docx]

Supplementary Table 2. Function of lncRNAs in EMT.

| **Cancer type** | **lncRNA** | **Numbers of clinical samples** | **Cell Line** | **Targets/ Regulators** | **Signaling Pathways** | **Function** | **References** |
| --- | --- | --- | --- | --- | --- | --- | --- |
| CC | NEAT1 | Human Protein Atlas database | HeLa, SiHa | miR-361, HSP90, E-cadherin,  N-cadherin, Vimentin | - | NEAT1 via the miR-361/HSP90 axis could mediate the Inhibition of EMT in CC. | (Xu et al., 2020) |
| OC | CCAT1 | 25 pairs of OC and ACTs | SKOV3, CaOV3, 293T | miR-490-3p, TGFβR1, claudin, E-cadherin, N-cadherin, Vimentin, MMP9 | - | CCAT1 via sponging miR-490-3p could upregulate TGFβR1 to promote TGFβ1-induced EMT of OC cells. | (Mu et al., 2018) |
| OC | PTAR | TCGA database | SKOV3, A2780,  OVCAR3 | miR-101-3p, ZEB1, of E-cadherin, Vimentin, FN1 | - | PTAR by binding miR-101-3p and regulating ZEB1 could promote EMT in SOC. | (Liang et al., 2018) |
| OC | FLVCR1-AS1 | 50 pairs of OC and ACTs | A2780, 3AO,PEO1, SKOV3, OVCAR3, OVCAR8, HOSEpiCs | miR-513, YAP1, Caspase-3, Bax, Bcl-2, E-cadherin, Vimentin, Snail | - | FLVCR1-AS1 by mediating miR-513/YAP1 axis could facilitate EMT in OC. | (Yan et al., 2019) |
| Cholangiocarcinoma (CCA) | LINC00261 | 50 pairs of CCA and ACTs | QBC939, RBE, HIBEC, Huh-28, HuCCT1, CCLP-1 | E-cadherin | - | Overexpression of LINC00261 could promote CCA metastasis via EMT process. | (Gao et al., 2020) |
| CCA | CCAT1 | 120 pairs of CCA and ACTs | HCCC-9810, H69, HUCCT1, QBC-939, RBE | miR-152, E-cadherin, Vimentin, N-cadherin | - | CCAT1 by suppressing miR-152 could promote migration, invasion, and EMT in intrahepatic CCA. | (Zhang et al., 2017b) |
| Gastric Cancer (GC) | TP73-AS1 | 76 pairs of GC and ACTs | GES1, SGC7901, BGC823, MGC803, AGS | Bcl-2, Caspase-3,  E-cadherin | - | Overexpression of TP73-AS1 could promote cell migration and invasion. | (Zhang et al., 2018b) |
| GC | DLX6-AS1 | - | AGS, HGC-27, SGC-7901, GES-1,  BGC-823 | MAP4K1, FUS, E-cadherin, Vimentin, N-cadherin | - | DLX6-AS1 through FUS-regulated MAP4K1 could promote cell proliferation, migration, and EMT in GC. | (Wu et al., 2020a) |
| GC | HRCEG | 18 pairs of GC and ACTs | MKN28, BGC823 | HDAC1, E-cadherin, Vimentin, Snail | - | HRCEG via HDAC1 could inhibit cells proliferation and EMT in GC. | (Wu et al., 2020b) |
| GC | SNHG7 | 162 pairs of GC and ACTs | MKN-45, GES-1,  SGC-7901, N87 | miR-34a, Snail,  E-cadherin, Vimentin | - | SNHG7 by regulating miR-34a-Snail-EMT axis could accelerate cell migration and invasion in GC. | (Zhang et al., 2020) |
| GC | HCP5 | 84 pairs of GC and ACTs | GES-1, SGC-7901, BGC-823, MKN-45, HGC27 | miR-27b-3p, Vimentin, E-cadherin, N-cadherin, | - | HCP5 by targeting miR-27b-3p could Regulate EMT-related markers in GC. | (Chen et al., 2020) |
| GC | CHRF | 103 pairs of GC and ACTs | HGC-27, BGC-823, SGC-7901, MKN-45, MKN-7, GES-1 | Vimentin, E-cadherin, N-cadherin | - | CHRF by regulating EMT could promote cell invasion and migration in GC. | (Gong et al., 2020) |
| GC | SNHG6 | 78 pairs of GC and ACTs | MGC-803, AGS, SGC-7901, BGC-823, GES-1 | miR-101-3p, ZEB1,  E-cadherin, β-catenin, N-cadherin, Vimentin | p27 | SNHG6 through suppression of p27 and sponging miR-101-3p could promote cell proliferation and EMT in GC. | (Yan et al., 2017) |
| GC | MEG-3 | - | HGC-27, BGC-823, GES-1 | E-cadherin | - | MEG-3 by regulating EMT could suppress GC cell growth, invasion, and migration. | (Jiao and Zhang 2019) |
| Colorectal Cancer (CRC) | CPS1-IT1 | 24 pairs of CRC and ACTs | LoVo, SW620, SW480, LS174T,  HCT116, HT29, HUVEC | HIF-1α, LC3-II, Beclin-1, N-cadherin, ZO-1, Vimentin,  E-cadherin, | - | CPS1-IT by blocking hypoxia-induced autophagy through suppression of HIF-1α could suppress metastasis and EMT in CRC. | (Zhang et al., 2018a) |
| CRC | LINC01133 | 219 pairs of CRC and ACTs | HT29, HCT8, LS513, SW620, HCT116 | E-cadherin, SRSF6, Fibronectin, Vimentin | - | Overexpression of LINC01133 by interacting with SRSF6 could inhibit EMT and metastasis in CRC. | (Kong et al., 2016) |
| CRC | HIF1A-AS2 | 92 pairs of CRC and ACTs | SW620, DLD-1,  HT-29, HCT116, FHC, NCM460, 293T | miR-129-5p, DNMT3A, E-cadherin, N-cadherin | - | HIF1A-AS2 by regulating the miR-129-5p/DNMT3A axis could positively affect the progression and EMT formation of CRC. | (Lin et al., 2018) |
| CRC | SNHG1 | 338 pairs of CRC and ACTs | SW480, HCT116, Lovo, CaCO-2,  HT29, CCC-HIE-2 | miR‐497, miR‐195‐5p, E-cadherin, Vimentin, N-cadherin | - | SNHG1 by targeting miR‐497/miR‐195‐5p could modify EMT underlying CRC exacerbation. | (Bai et al., 2020) |
| CRC | SNHG6 | 29 pairs of CRC and ACTs | SW480, SW620,  HCT8, HT-29, NCM460 | miR-26a, EZH2, E-cadherin, Vimentin, N-cadherin, Snail |  | SNHG6 via the miR-26a/EZH2 axis could promote the migration, invasion, and EMT of CRC cells. | (Zhang et al., 2019a) |
| CRC | PANDAR | 124 pairs of CRC and ACTs | SW480, LoVo, HCT-116, SW620, HT29, HcoEpic | Bcl-2, Bax, Snail, Twist, E-cadherin, N-cadherin, Vimentin,  β-catenin | - | Overexpression of PANDAR by EMT pathway could promote metastasis in CRC. | (Lu et al., 2017) |
| CRC | LINC01413 | 3 pairs of CRC and ACTs | SW480, SW620, HCT-116, HT-29, LoVo, NCM460 | hnRNP-K, ZEB1, YAP1, E-cadherin, TAZ1, N-cadherin, | - | LINC01413/hnRNP-K/ZEB1 axis via inducing YAP1/TAZ1 translocation could accelerate EMT. | (Ji et al., 2020a) |
| Triple-Negative Breast Cancer (TNBC) | XIST | 35 pairs of TNBC and ACTs | MDA-MB-468, MDAMB-231, MCF-10A | miR-454, E-cadherin, N-cadherin, Snail, Vimentin | - | XIST by targeting miR-454 could inhibit cells proliferation, and EMT in TNBC. | (Li et al., 2020) |
| Breast Cancer (BC) | MIF-AS1 | 82 pairs of BC and ACTs | MCF-10A, MCF-7, MDA-MB-231, MDA-MB-468 | miR-1249-3p, HOXB8, E-cadherin, N-cadherin, Slug, Snail, Vimentin, Fibronectin | - | MIF-AS1 through regulating the miR-1249-3p/HOXB8 axis could promote EMT process. | (Ding et al., 2019) |
| BC | UCA1 | - | MDA-MB-231 | E-cadherin, MMP-7,  N-cadherin, Snail, Vimentin, Cyclin D1, | Wnt/β-catenin | UCA1 via enhancing the Wnt/β-catenin signaling pathway could promote EMT of BC cells. | (Xiao et al., 2016) |
| BC | LINC00665 | GEPIA database, 60 pairs of BC and ACTs | MCF-7, MDA-MB-231, ZR-75-30, MCF-10A, MDA-MB-415 | E-cadherin, Vimentin, N-cadherin, β-catenin, | - | LINC00665 by triggering EMT could promote metastasis of BC cells. | (Zhou et al., 2020) |
| BC | H19 | 60 pairs of BC and ACTs | 168FARN, 4TO7, 4T1, Scp2, TA2-C13, TA2-C47 | miR-200b/c, let-7b, GIT2, N-cadherin, E-cadherin | - | H19 by sponging miR-200b/c and let-7b could mediate BC cell plasticity during EMT and MET plasticity. | (Zhou et al., 2017) |
| BC | HOXA11-AS | 68 pairs of BC and ACTs | MDA-MB-231, MDA-MB-468, MDA-MB-435, SKBR3, MCF-7,  MCF-10A | E-cadherin, N-cadherin, Vimentin | - | HOXA11-AS by regulating EMT could promote BC invasion and metastasis. | (Li et al., 2017) |
| Prostate Cancer (PCa) | SNHG7 | 499 pairs of PCa and ACTs | RWPE, LNCaP,  PC-3, Du-145, 293T | miR-324-3p, WNT2B, N-cadherin, Snail, E-cadherin, Vimentin, Slug, Mmp-2, Mmp-9, Twist-1 | - | Knockdown of SNHG7 via the miR-324-3p/WNT2B axis could inhibit EMT  in PCa. | (Hu 2019) |
| PCa | PVT1 | TCGA database | PC-3, DU145, 22RV1, WPMY | Twist-1, Vimentin, Snail, Slug, E-cadherin, miR-186-5p | - | PVT1 via mediating the miR-186-5p/Twist-1 axis could promote EMT in PCa. | (Chang et al., 2018) |
| Hepatic Carcinoma (HC) | SNHG7 | 40 pairs of HC and ACTs | HepG2, HCC‐LM3 | miR‐425, N‐cadherin, E-cadherin, MMP-9 | Wnt/β‐catenin/EMT | SNHG7 by sponging miR‐425 via the Wnt/β‐catenin/EMT could promote proliferation, migration, and invasiveness of HC. | (Yao et al., 2019) |
| HCC | SBF2-AS1 | 134 pairs of HCC and ACTs | HCCLM3, Huh7, SK-Hep1, HepG2, L-02 | E-cadherin, N-cadherin, Vimentin | - | SBF2-AS1 by regulating EMT could promote HCC metastasis. | (Zhang et al., 2018d) |
| HCC | LINC00668 | 40 pairs of HCC and ACTs | HepG2, SNU-387, MHCC-97H, Huh-7, THLE-3 | miR-532-5p, YY1, E-cadherin, Vimentin, N-cadherin | - | LINC00668 through modulating the miR-532-5p/YY1 axis could promote cell proliferation, migration, invasion, and EMT in HCC. | (Xuan et al., 2020) |
| HCC | LOC105372579 | 54 pairs of HCC and ACTs | SMCC7721, HepG2, Hep3B, Huh7, L-02 | miR-4316, FOXP4,  E-cadherin, N-cadherin | - | LOC105372579 via activating miR-4316/FOXP4 signaling could promote proliferation and EMT in HCC. | (E et al., 2019) |
| HCC | CRNDE | 12 pairs of HCC and ACTs | HepG2, L-02, SMMC7721,  SK-hep1, Huh7 | E-cadherin, ZO-1, N-cadherin, Slug, Twist, Vimentin, Frizzled-4 | Wnt/β‐catenin | CRNDE via enhancing the Wnt/β‐catenin could promote the EMT of HCC cells. | (Zhu et al., 2018) |
| HCC | HULC | 38 HCC samples and 21 normal liver samples | Huh-6, Huh-7,  HepG2, BEL-7402, MHCC-97H,  Sk-Hep1, L-02,  SMMC-7721 | miR-200a-3p, ZEB1, N-Cadherin, Vimentin, ZO-1, E-cadherin, Snail | - | HULC via the miR-200a-3p/ZEB1 signaling pathway could enhance EMT to promote tumorigenesis and metastasis of HCC. | (Li et al., 2016) |
| HCC | HOXA‐AS3 | 76 pairs of HCC and ACTs | SMMC-7721, HepG2, Huh7 HCCLM3, L-02 | miR‐29c, E-cadherin, N-cadherin, Vimentin | MEK/ERK | HOXA‐AS3 by sponging miR‐29c and activating the MEK/ERK signaling pathway could facilitate cell proliferation, metastasis, and EMT process in HCC. | (Tong et al., 2019) |
| HCC | SNHG3 | - | PLC/PRF/5, Hep3B, HepG2, MHCC97L, Huh7, SMMC-7721, HCCLM3 | miR-128, CD151,  N-Cadherin, Vimentin, E-cadherin, Snail | - | SNHG3 by modulating the miR‐128/CD151 axis could induce EMT and sorafenib resistance in HCC. | (Zhang et al., 2019b) |
| Osteosarcoma (OS) | FER1L4 | 35 pairs of OS and ACTs | hFOB1.19, MG63,  U2OS, HOS, Saos-2 | SOCS5, miR-18a-5p, Caspase-3, Bax, Bcl-2, Twist1, N-cadherin, Vimentin, Nanog | PI3K/Akt | FER1L4 by suppressing miR-18a-5p induces apoptosis and suppresses EMT in OS cells. | (Ye et al., 2019) |
| OS | CRNDE | 8 pairs of OS and ACTs | hFOB1.19, U2OS, MG63, MNNG/HOS | E-cadherin, Vimentin, ZO-1, N-cadherin, Snail, GSK‐3β | Wnt/β‐catenin | CRNDE is induced by SP1 and promotes OS proliferation, invasion, and EMT. | (Ding et al., 2020) |
| Oral Squamous Cell Carcinoma (OSCC) | ADAMTS9-AS2 | 76 pairs of OSCC and ACTs | Cal27, SCC9, SCC15, SCC25, SCC4, NOKs | E-cadherin, EZH2,  N-cadherin, miR-600 | - | ADAMTS9-AS2 via the miR-600/EZH2 axis could promote OSCC proliferation, migration, and EMT. | (Li et al., 2019) |
| OSCC | H19 | 123 OSCC tissues and 50 ACTs | Cal27, SCC9, SCC15, SCC25, Tca8113, UM1 | E-cadherin, Vimentin, ZO-1, N-cadherin, GSK-3β, Cyclin D1,  c-myc, EZH2, β-catenin | - | H19 via the β-catenin/GSK3β/EMT signaling via association with EZH2 could promote OSCC progression. | (Zhang et al., 2017a) |
| OSCC | MALAT1 | 123 OSCC tissues, 50 ACTs | CAL-27, SCC-9, CAL-27/DDP,  SCC-9/DDP | E-cadherin, P-gp, N-cadherin, | PI3K/Akt/mTOR | MALAT1 via the PI3K/AKT/mTOR signaling pathway could promote the EMT process and cisplatin resistance of OSCC. | (Wang et al., 2020) |
| Pancreatic Cancer (PaC) | XIST | 120 pairs of PaC and ACTs | ASPC-1, PANC-1, HPAC, BxPC-3, CFPAC-1, HPDE | miR-429, ZEB-1, E-cadherin, Claudin-1, β-catenin, Snail | - | XIST by sponging miR-429 to modulate ZEB1 expression could promote PaC migration, invasion, and EMT. | (Shen et al., 2019) |
| PaC | TUG1 | 30 pairs of PaC and ACTs | SW1990, BxPC3, PaTu8988 | MMP2, MMP9,  E-cadherin | TGF-β/Smad | TUG1 via EMT pathway could promote proliferation and migration of PaC. | (Qin and Zhao 2017) |
| PaC | PVT1 | 20 pairs of PaC and ACTs | PANC-1, HPDE6c7 | Snail, ZEB1 | p21 | PVT1 by downregulating p21 could promote EMT and cell proliferation and migration in PaC cells. | (Wu et al., 2017) |
| PaC | TUG1 | 34 pairs of PaC and ACTs | PANC-1, AsPC-1, 293T, PATU 8988, BxPC-3, SW1990, HPDE6-C7 | EZH2, E-cadherin, N-cadherin, Vimentin, β‐catenin | - | TUG1 via miR-382/EZH2 axis could promote PaC cell proliferation, migration, and EMT phenotype formation. | (Zhao et al., 2017) |
| PaC | SNHG12 | 15 pairs of PaC and ACTs | HPDE6, BXPC3, CAPAN1, PANC1, SW1990 | miR-320b, E-cadherin,  N-cadherin, Vimentin | - | SNHG12 by targeting miR-320b could increase proliferation, invasion, and EMT of PaC cells. | (Cao and Zhou 2020) |
| Bladder Cancer (BLC) | TP73-AS1 | 128 pairs of BLC and ACTs | T24, 5637, SW780, BIU87, J82, HT1376 | Vimentin, Snail, MMP-2, MMP-9, E-cadherin, Caspase-3 | - | TP73-AS1 by the inactivation of the EMT pathway could inhibit BLC cell proliferation, migration, and invasion. | (Tuo et al., 2018) |
| BLC | NRON | 42 pairs of BLC and ACTs | J82, 5637, T24, UMUC3, SW780, | Vimentin, N-cadherin, E-cadherin, EZH2 | - | NRON by targeting EZH2 could promote the proliferation, metastasis and EMT process in BLC. | (Xiong et al., 2020) |
| BLC | UCA1 | 42 pairs of BLC and ACTs | T24, 5637, J82, RT4, HT1376 | miR-143, HMGB1, Vimentin, N-cadherin, E-cadherin | - | UCA1 by regulating the miR‑143/HMGB1 pathway could promote the invasion and EMT of BLC cells. | (Luo et al., 2017) |
| Nasopharyngeal carcinoma (NPC) | TUG1 | 48 pairs of NPC and ACTs | CNE1, CNE2, HONE1, HNE1, C666e1, S26  S18, NP69 | miR-384, Vimentin,  N-cadherin, TGF-β1, E-cadherin | - | Knockdown of TUG1 by inhibiting EMT via the promotion of miR-384 could suppress NPC progression. | (Qian et al., 2019) |
| Retinoblastoma (RB) | XIST | 35 human RB samples and 7 normal retina samples | SO-RB50, Y79,  Weri-Rb1, ARPE-19 | miR-101, ZEB1, ZEB2, E-cadherin, Vimentin, Caspase-3 | - | XIST via sponging miR-101 could promote the EMT of RB. | (Cheng et al., 2019) |
| Osteosarcoma (OS) | CRNDE | 48 pairs of OS and ACTs | hFOB, MG-63, SAOS-2, U2OS | Notch1, JAG1,  N-cadherin, Vimentin,  E-cadherin | - | CRNDE by regulating Notch1 could promote osteosarcoma cell proliferation, invasion, migration, and EMT. | (Li et al., 2018) |
| OS | PCAT1 | 30 pairs of OS and ACTs | NHost, LM7, KHOS, MG-63, U2OS | Vimentin, E-cadherin, N-cadherin | - | PCAT1 could promote cell proliferation, invasion, migration, and EMT in OS. | (Zhang et al., 2018c) |
| Lung Adenocarcinoma (LUAD) | JPX | 116 pairs of LUAD and ACTs | BEAS-2B, SPC-A-1, LTEP-a-2, A549, NCI-H1299 | miR-33a-5p, Twist1, Vimentin, E-cadherin, N-cadherin, GSK-3β | Wnt/β-catenin | JPX/miR-33a-5p/Twist1 axis could induce lung cancer cell EMT progression. | (Pan et al., 2020) |
| LUAD | H19 | 305 pairs of LUAD and ACTs | Calu-3, NCI-H1975, A549, NCI-H23, HLF-a | miR-29b-3p, STAT3, Vimentin, E-cadherin, Slug, Snail | - | H19 by affecting miR-29b-3p and STAT3 expressions could promote the viability and EMT of LUAD cells. | (Liu et al., 2019) |
| LUAD | TTN-AS1 | 107 pairs of LUAD and ACTs | H1650, HCC827, A549, H1975,  PC9, BEAS-2B | miR-142-5p, CDK5, Vimentin, E-cadherin, Twist, Snail | - | TTN-AS1 by sponging miR-142-5p to regulate CDK5 could promote migration, invasion, and EMT of LUAD. | (Jia et al., 2019) |
| Non-Small Cell Lung Carcinoma (NSCLC) | DUXAP8 | 54 pairs of tumors and ACTs | H460, H520, A549, H1975, BEAS-2B | miR-498, TRIM44,  N-cadherin, E-cadherin, Vimentin | Akt/mTOR | Downregulation of DUXAP8 by influencing miR-498 via TRIM44-mediated AKT/mTOR pathway could suppress proliferation, metastasis, and EMT in NSCLC. | (Ji et al., 2020b) |
| NSCLC | NBR2 | 50 pairs of tumors and ACTs | BEAS2B, A549, AsPC-1, H460 | N-cadherin, E-cadherin, Vimentin, Notch1, HEY1, HEY2, HEYL | - | NBR2 by regulating Notch1 could inhibit EMT progression in NSCLC. | (Gao et al., 2019) |
| NSCLC | FEZF1-AS1 | 86 pairs of tumors and ACTs | A549, SPC-A1, H1299, H1975,  PC9, 16HBE | Slug, twist, E-cadherin, ZO-1, Vimentin, EZH2, LSD1 | Wnt/β-catenin | FEZF1-AS1 by regulating the WNT pathway and suppressing E-cadherin could enhance EMT in NSCLC. | (He et al., 2017) |
| NSCLC | MALAT1 | 86 pairs of tumors and ACTs | NSCLC A549, NCI-H460, NCI-H529, SK-MES-1, 16HBE | miR-124, E-cadherin, Vimentin | - | MALAT-1 by regulating miR-124 could promote EMT and development of NSCLC. | (Wu et al., 2018) |
| Esophageal Squamous Cell Carcinoma  (ESCC) | NR2F1-AS1 | 51 pairs of ESCC and ACTs | ECA109, TE-1,  ECA7906, KYSE-30, KYSE-70, Het-1A | E-cadherin, Vimentin,  N-cadherin, | - | NR2F1-AS1 by regulating EMT could promote proliferation and metastasis of ESCC cells. | (Ren et al., 2020) |
| ESCC | GHET1 | 55 pairs of ESCC and ACTs | EC109, EC9706, KYSE30, KYSE450, Het-1A | N-cadherin, Vimentin, E-cadherin | - | GHET1 via induction of EMT could promote ESCC cell proliferation and invasion. | (Liu et al., 2017) |
| ESCC | BDNF-AS | 54 pairs of ESCC and ACTs | SHEE, OE19, KYSE-70, KYSE-170, KYSE-180, OE33, Eca-109, TE-1,  TE-13 | miR-214, N-cadherin, Vimentin, E-cadherin | - | BDNF-AS by targeting miR-214 could inhibit proliferation, migration, invasion, and EMT in ESCC cells. | (Zhao et al., 2018) |
| ESCC | MALAT1 | - | KYSE30, OE21, EC109, TE-1,  Het-1A | Ezh2, Notch1, Hes1, MMP-9, Vimentin, E-cadherin | - | MALAT1 through the Ezh2/Notch1 axis could promote EMT of ESCC. | (Chen et al., 2018) |
| Head and Neck Squamous Cell Carcinoma (HNSCC) | LINC00460 | 123 pairs of ESCC and ACTs | WSU-HN4, HeLa, WSUHN6, A549,  WSU-HN30, SCC-4, SCC-9, SCC-25,  CAL-27 | E-cadherin, PRDX1, N-cadherin, Vimentin, ZEB1, ZEB2 | - | LINC00460 by facilitating PRDX1 into the nucleus could promote EMT in HNSCC. | (Jiang et al., 2019) |
| Papillary Thyroid  Cancer (PTC) | TUG1 | 36 pairs of PTC and ACTs | SW1736, KAT18, FTC, FTC133,  HGC-27 | miR-145, ZEB1, N-cadherin, E-cadherin | - | TUG1 by targeting miR-145 could influence PTC cell proliferation, migration, and EMT formation. | (Lei et al., 2017) |
| PTC | BANCR | 27 pairs of PTC and ACTs | BCPAP, CAL‑62, WRO, FTC‑133 | N-cadherin, Vimentin, E-cadherin | Raf/MEK/ERK | BANCR via the Raf/MEK/ERK signaling pathway could promote EMT in PTC. | (Wang et al., 2018) |
| Glioblastoma (GBM) | H19 | 20 pairs of GBM and ACTs | U87, U251, A172, LN229, U118, NHAs | N-cadherin, Vimentin, miR-130a-3p | - | H19 by sponging miR-130a-3p could regulate EMT in GBM. | (Hu et al., 2018) |
| Renal Cell Carcinoma (RCC) | PVT1 | 25 pairs of RCC and ACTs | A498, 786-O, HK-2, ACHN, Caki-1 | miR-16-5p, Vimentin,  Bax, Bcl-2, Bcl-XL, Caspase-3,  N-cadherin, E-cadherin | - | PVT1 by downregulating miR-16-5p enhances proliferation, invasion, and EMT of RCC cells. | (Ren et al., 2019) |

**References**

Bai, J., Xu, J., Zhao, J. and Zhang, R. (2020). lncRNA SNHG1 cooperated with miR‐497/miR‐195‐5p to modify epithelial–mesenchymal transition underlying colorectal cancer exacerbation. Journal of cellular physiology 235 1453-1468.

Cao, W. and Zhou, G. (2020). LncRNA SNHG12 contributes proliferation, invasion and epithelial-mesenchymal transition of pancreatic cancer cells by absorbing miRNA-320b. Biosci Rep 40.

Chang, Z., Cui, J. and Song, Y. (2018). Long noncoding RNA PVT1 promotes EMT via mediating microRNA-186 targeting of Twist1 in prostate cancer. Gene 654 36-42.

Chen, M., Xia, Z., Chen, C., Hu, W. and Yuan, Y. (2018). LncRNA MALAT1 promotes epithelial-to-mesenchymal transition of esophageal cancer through Ezh2-Notch1 signaling pathway. Anticancer Drugs 29 767-773.

Chen, S., Ren, C., Zheng, H., Sun, X. and Dai, J. (2020). The Effect of Long Non-Coding RNA (lncRNA) HCP5 on Regulating Epithelial-Mesenchymal Transition (EMT)-Related Markers in Gastric Carcinoma Is Partially Reversed by miR-27b-3p. Med Sci Monit 26 e921383.

Cheng, Y., Chang, Q., Zheng, B., Xu, J., Li, H. and Wang, R. (2019). LncRNA XIST promotes the epithelial to mesenchymal transition of retinoblastoma via sponging miR-101. European journal of pharmacology 843 210-216.

Ding, J., Wu, W., Yang, J. and Wu, M. (2019). Long non-coding RNA MIF-AS1 promotes breast cancer cell proliferation, migration and EMT process through regulating miR-1249-3p/HOXB8 axis. Pathol Res Pract 215 152376.

Ding, Q., Mo, F., Cai, X., Zhang, W., Wang, J., Yang, S. and Liu, X. (2020). LncRNA CRNDE is activated by SP1 and promotes osteosarcoma proliferation, invasion, and epithelial‐mesenchymal transition via Wnt/β‐catenin signaling pathway. J Cell Biochem.

E, C., Yang, J., Li, H. and Li, C. (2019). LncRNA LOC105372579 promotes proliferation and epithelial-mesenchymal transition in hepatocellular carcinoma via activating miR-4316/FOXP4 signaling. Cancer Manag Res 11 2871-2879.

Gao, J., Qin, W., Kang, P., Xu, Y., Leng, K., Li, Z., et al. (2020). Up-regulated LINC00261 predicts a poor prognosis and promotes a metastasis by EMT process in cholangiocarcinoma. Pathology-Research and Practice 216 152733.

Gao, Y., Li, Y., Li, H. and Zhao, B. (2019). LncRNA NBR2 inhibits EMT progression by regulating Notch1 pathway in NSCLC. European Review for Medical and Pharmacological Sciences 23 7950-7958.

Gong, J., Wang, Y. and Shu, C. (2020). LncRNA CHRF promotes cell invasion and migration via EMT in gastric cancer. Eur Rev Med Pharmacol Sci 24 1168-1176.

He, R., Zhang, F. H. and Shen, N. (2017). LncRNA FEZF1-AS1 enhances epithelial-mesenchymal transition (EMT) through suppressing E-cadherin and regulating WNT pathway in non-small cell lung cancer (NSCLC). Biomed Pharmacother 95 331-338.

Hu, H. (2019). Knockdown of LncRNA SNHG7 inhibited epithelial-mesenchymal transition in prostate cancer though miR-324-3p/WNT2B axis in vitro. Pathology-Research and Practice 215 152537.

Hu, Q., Yin, J., Zeng, A., Jin, X., Zhang, Z., Yan, W. and You, Y. (2018). H19 Functions as a Competing Endogenous RNA to Regulate EMT by Sponging miR-130a-3p in Glioma. Cell Physiol Biochem 50 233-245.

Ji, L., Li, X., Zhou, Z., Zheng, Z., Jin, L. and Jiang, F. (2020a). LINC01413/hnRNP-K/ZEB1 Axis Accelerates Cell Proliferation and EMT in Colorectal Cancer via Inducing YAP1/TAZ1 Translocation. Molecular Therapy. Nucleic Acids 19 546.

Ji, X., Tao, R., Sun, L. Y., Xu, X. L. and Ling, W. (2020b). Down-regulation of long non-coding RNA DUXAP8 suppresses proliferation, metastasis and EMT by modulating miR-498 through TRIM44-mediated AKT/mTOR pathway in non-small-cell lung cancer. Eur Rev Med Pharmacol Sci 24 3152-3165.

Jia, Y., Duan, Y., Liu, T., Wang, X., Lv, W., Wang, M., et al. (2019). LncRNA TTN-AS1 promotes migration, invasion, and epithelial mesenchymal transition of lung adenocarcinoma via sponging miR-142-5p to regulate CDK5. Cell Death Dis 10 573.

Jiang, Y., Cao, W., Wu, K., Qin, X., Wang, X., Li, Y., et al. (2019). LncRNA LINC00460 promotes EMT in head and neck squamous cell carcinoma by facilitating peroxiredoxin-1 into the nucleus. Journal of Experimental & Clinical Cancer Research 38 365.

Jiao, J. and Zhang, S. (2019). Long non‑coding RNA MEG‑3 suppresses gastric carcinoma cell growth, invasion and migration via EMT regulation. Molecular medicine reports 20 2685-2693.

Kong, J., Sun, W., Li, C., Wan, L., Wang, S., Wu, Y., et al. (2016). Long non-coding RNA LINC01133 inhibits epithelial–mesenchymal transition and metastasis in colorectal cancer by interacting with SRSF6. Cancer letters 380 476-484.

Lei, H., Gao, Y. and Xu, X. (2017). LncRNA TUG1 influences papillary thyroid cancer cell proliferation, migration and EMT formation through targeting miR-145. Acta Biochim Biophys Sin (Shanghai) 49 588-597.

Li, S.-P., Xu, H.-X., Yu, Y., He, J.-D., Wang, Z., Xu, Y.-J., et al. (2016). LncRNA HULC enhances epithelial-mesenchymal transition to promote tumorigenesis and metastasis of hepatocellular carcinoma via the miR-200a-3p/ZEB1 signaling pathway. Oncotarget 7 42431.

Li, W., Jia, G., Qu, Y., Du, Q., Liu, B. and Liu, B. (2017). Long non-coding RNA (LncRNA) HOXA11-AS promotes breast cancer invasion and metastasis by regulating epithelial-mesenchymal transition. Medical science monitor: international medical journal of experimental and clinical research 23 3393.

Li, X., Hou, L., Yin, L. and Zhao, S. (2020). LncRNA XIST interacts with miR-454 to inhibit cells proliferation, epithelial mesenchymal transition and induces apoptosis in triple-negative breast cancer. J Biosci 45.

Li, Y., Wan, Q., Wang, W., Mai, L., Sha, L., Mashrah, M., et al. (2019). LncRNA ADAMTS9-AS2 promotes tongue squamous cell carcinoma proliferation, migration and EMT via the miR-600/EZH2 axis. Biomedicine & Pharmacotherapy 112 108719.

Li, Z., Tang, Y., Xing, W., Dong, W. and Wang, Z. (2018). LncRNA, CRNDE promotes osteosarcoma cell proliferation, invasion and migration by regulating Notch1 signaling and epithelial-mesenchymal transition. Experimental and molecular pathology 104 19-25.

Liang, H., Yu, T., Han, Y., Jiang, H., Wang, C., You, T., et al. (2018). LncRNA PTAR promotes EMT and invasion-metastasis in serous ovarian cancer by competitively binding miR-101-3p to regulate ZEB1 expression. Molecular cancer 17 1-13.

Lin, J., Shi, Z., Yu, Z. and He, Z. (2018). LncRNA HIF1A-AS2 positively affects the progression and EMT formation of colorectal cancer through regulating miR-129-5p and DNMT3A. Biomedicine & Pharmacotherapy 98 433-439.

Liu, H., Zhen, Q. and Fan, Y. (2017). LncRNA GHET1 promotes esophageal squamous cell carcinoma cells proliferation and invasion via induction of EMT. The International journal of biological markers 32 403-408.

Liu, L., Liu, L. and Lu, S. (2019). lncRNA H19 promotes viability and epithelial-mesenchymal transition of lung adenocarcinoma cells by targeting miR-29b-3p and modifying STAT3. Int J Oncol 54 929-941.

Lu, M., Liu, Z., Li, B., Wang, G., Li, D. and Zhu, Y. (2017). The high expression of long non-coding RNA PANDAR indicates a poor prognosis for colorectal cancer and promotes metastasis by EMT pathway. Journal of cancer research and clinical oncology 143 71-81.

Luo, J., Chen, J., Li, H., Yang, Y., Yun, H., Yang, S. and Mao, X. (2017). LncRNA UCA1 promotes the invasion and EMT of bladder cancer cells by regulating the miR‑143/HMGB1 pathway. Oncology letters 14 5556-5562.

Mu, Y., Li, N. and Cui, Y.-L. (2018). The lncRNA CCAT1 upregulates TGFβR1 via sponging miR-490-3p to promote TGFβ1-induced EMT of ovarian cancer cells. Cancer cell international 18 145.

Pan, J., Fang, S., Tian, H., Zhou, C., Zhao, X., Tian, H., et al. (2020). lncRNA JPX/miR-33a-5p/Twist1 axis regulates tumorigenesis and metastasis of lung cancer by activating Wnt/β-catenin signaling. Molecular cancer 19 1-17.

Qian, W., Ren, Z. and Lu, X. (2019). Knockdown of long non-coding RNA TUG1 suppresses nasopharyngeal carcinoma progression by inhibiting epithelial-mesenchymal transition (EMT) via the promotion of miR-384. Biochemical and biophysical research communications 509 56-63.

Qin, C. and Zhao, F. (2017). Long non-coding RNA TUG1 can promote proliferation and migration of pancreatic cancer via EMT pathway. Eur Rev Med Pharmacol Sci 21 2377-2384.

Ren, P., Zhang, H., Chang, L., Hong, X. and Xing, L. (2020). LncRNA NR2F1-AS1 promotes proliferation and metastasis of ESCC cells via regulating EMT. European Review for Medical and Pharmacological Sciences 24 3686-3693.

Ren, Y., Huang, W., Weng, G., Cui, P., Liang, H. and Li, Y. (2019). LncRNA PVT1 promotes proliferation, invasion and epithelial-mesenchymal transition of renal cell carcinoma cells through downregulation of miR-16-5p. Onco Targets Ther 12 2563-2575.

Shen, J., Hong, L., Yu, D., Cao, T., Zhou, Z. and He, S. (2019). LncRNA XIST promotes pancreatic cancer migration, invasion and EMT by sponging miR-429 to modulate ZEB1 expression. The international journal of biochemistry & cell biology 113 17-26.

Tong, Y., Wang, M., Dai, Y., Bao, D., Zhang, J. and Pan, H. (2019). LncRNA HOXA-AS3 sponges miR-29c to facilitate cell proliferation, metastasis, and EMT process and activate the MEK/ERK signaling pathway in hepatocellular carcinoma. Human Gene Therapy Clinical Development 30 129-141.

Tuo, Z., Zhang, J. and Xue, W. (2018). LncRNA TP73-AS1 predicts the prognosis of bladder cancer patients and functions as a suppressor for bladder cancer by EMT pathway. Biochemical and biophysical research communications 499 875-881.

Wang, R., Lu, X. and Yu, R. (2020). lncRNA MALAT1 Promotes EMT Process and Cisplatin Resistance of Oral Squamous Cell Carcinoma via PI3K/AKT/m-TOR Signal Pathway. OncoTargets and therapy 13 4049.

Wang, Y., Gu, J., Lin, X., Yan, W., Yang, W. and Wu, G. (2018). lncRNA BANCR promotes EMT in PTC via the Raf/MEK/ERK signaling pathway. Oncology letters 15 5865-5870.

Wu, B.-Q., Jiang, Y., Zhu, F., Sun, D.-L. and He, X.-Z. (2017). Long noncoding RNA PVT1 promotes EMT and cell proliferation and migration through downregulating p21 in pancreatic cancer cells. Technology in cancer research & treatment 16 819-827.

Wu, J., Weng, Y., He, F., Liang, D. and Cai, L. (2018). LncRNA MALAT-1 competitively regulates miR-124 to promote EMT and development of non-small-cell lung cancer. Anti-cancer drugs 29 628-636.

Wu, Q., Ma, J., Meng, W. and Hui, P. (2020a). DLX6-AS1 promotes cell proliferation, migration and EMT of gastric cancer through FUS-regulated MAP4K1. Cancer biology & therapy 21 17-25.

Wu, S., Wu, E., Wang, D., Niu, Y., Yue, H., Zhang, D., et al. (2020b). LncRNA HRCEG, regulated by HDAC1, inhibits cells proliferation and epithelial-mesenchymal-transition in gastric cancer. Cancer Genet 241 25-33.

Xiao, C., Wu, C. and Hu, H. (2016). LncRNA UCA1 promotes epithelial-mesenchymal transition (EMT) of breast cancer cells via enhancing Wnt/beta-catenin signaling pathway. Eur Rev Med Pharmacol Sci 20 2819-2824.

Xiong, T., Huang, C., Li, J., Yu, S., Chen, F., Zhang, Z., et al. (2020). LncRNA NRON promotes the proliferation, metastasis and EMT process in bladder cancer. J Cancer 11 1751-1760.

Xu, D., Dong, P., Xiong, Y., Yue, J., Konno, Y., Ihira, K., et al. (2020). MicroRNA-361-Mediated Inhibition of HSP90 Expression and EMT in Cervical Cancer Is Counteracted by Oncogenic lncRNA NEAT1. Cells 9.

Xuan, W., Zhou, C. and You, G. (2020). LncRNA LINC00668 promotes cell proliferation, migration, invasion ability and EMT process in hepatocellular carcinoma by targeting miR-532-5p/YY1 axis. Biosci Rep 40.

Yan, H., Li, H., Silva, M. A., Guan, Y., Yang, L., Zhu, L., et al. (2019). LncRNA FLVCR1-AS1 mediates miR-513/YAP1 signaling to promote cell progression, migration, invasion and EMT process in ovarian cancer. Journal of Experimental & Clinical Cancer Research 38 1-13.

Yan, K., Tian, J., Shi, W., Xia, H. and Zhu, Y. (2017). LncRNA SNHG6 is associated with poor prognosis of gastric cancer and promotes cell proliferation and EMT through epigenetically silencing p27 and sponging miR-101-3p. Cellular Physiology and Biochemistry 42 999-1012.

Yao, X., Liu, C., Liu, C., Xi, W., Sun, S. and Gao, Z. (2019). lncRNA SNHG7 sponges miR‐425 to promote proliferation, migration, and invasion of hepatic carcinoma cells via Wnt/β‐catenin/EMT signalling pathway. Cell biochemistry and function 37 525-533.

Ye, F., Tian, L., Zhou, Q. and Feng, D. (2019). LncRNA FER1L4 induces apoptosis and suppresses EMT and the activation of PI3K/AKT pathway in osteosarcoma cells via inhibiting miR-18a-5p to promote SOCS5. Gene 721 144093.

Zhang, D.-M., Lin, Z.-Y., Yang, Z.-H., Wang, Y.-Y., Wan, D., Zhong, J.-L., et al. (2017a). IncRNA H19 promotes tongue squamous cell carcinoma progression through β-catenin/GSK3β/EMT signaling via association with EZH2. American journal of translational research 9 3474.

Zhang, M., Duan, W. and Sun, W. (2019a). LncRNA SNHG6 promotes the migration, invasion, and epithelial-mesenchymal transition of colorectal cancer cells by miR-26a/EZH2 axis. OncoTargets and therapy 12 3349.

Zhang, P. F., Wang, F., Wu, J., Wu, Y., Huang, W., Liu, D., et al. (2019b). LncRNA SNHG3 induces EMT and sorafenib resistance by modulating the miR‐128/CD151 pathway in hepatocellular carcinoma. Journal of cellular physiology 234 2788-2794.

Zhang, S., Xiao, J., Chai, Y., yan Du, Y., Liu, Z., Huang, K., et al. (2017b). LncRNA-CCAT1 promotes migration, invasion, and EMT in intrahepatic cholangiocarcinoma through suppressing miR-152. Digestive diseases and sciences 62 3050-3058.

Zhang, W., Yuan, W., Song, J., Wang, S. and Gu, X. (2018a). LncRNA CPS1-IT1 suppresses EMT and metastasis of colorectal cancer by inhibiting hypoxia-induced autophagy through inactivation of HIF-1α. Biochimie 144 21-27.

Zhang, W., Zhai, Y., Wang, W., Cao, M. and Ma, C. (2018b). Enhanced expression of lncRNA TP73-AS1 predicts unfavorable prognosis for gastric cancer and promotes cell migration and invasion by induction of EMT. Gene 678 377-383.

Zhang, X., Zhang, Y., Mao, Y. and Ma, X. (2018c). The lncRNA PCAT1 is correlated with poor prognosis and promotes cell proliferation, invasion, migration and EMT in osteosarcoma. Onco Targets Ther 11 629-638.

Zhang, Y., Li, B., Zhang, B., Ma, P., Wu, Q., Ming, L. and Xie, L. (2018d). LncRNA SBF2-AS1 promotes hepatocellular carcinoma metastasis by regulating EMT and predicts unfavorable prognosis. Eur Rev Med Pharmacol Sci 22 6333-6341.

Zhang, Y., Yuan, Y., Zhang, Y., Cheng, L., Zhou, X. and Chen, K. (2020). SNHG7 accelerates cell migration and invasion through regulating miR-34a-Snail-EMT axis in gastric cancer. Cell Cycle 19 142-152.

Zhao, H., Diao, C., Wang, X., Xie, Y., Liu, Y., Gao, X., et al. (2018). LncRNA BDNF-AS inhibits proliferation, migration, invasion and EMT in oesophageal cancer cells by targeting miR-214. J Cell Mol Med 22 3729-3739.

Zhao, L., Sun, H., Kong, H., Chen, Z., Chen, B. and Zhou, M. (2017). The Lncrna-TUG1/EZH2 Axis Promotes Pancreatic Cancer Cell Proliferation, Migration and EMT Phenotype Formation Through Sponging Mir-382. Cell Physiol Biochem 42 2145-2158.

Zhou, J., Zou, L. and Zhu, T. (2020). Long non-coding RNA LINC00665 promotes metastasis of breast cancer cells by triggering EMT. European Review for Medical and Pharmacological Sciences 24 3097-3104.

Zhou, W., Ye, X. L., Xu, J., Cao, M. G., Fang, Z. Y., Li, L. Y., et al. (2017). The lncRNA H19 mediates breast cancer cell plasticity during EMT and MET plasticity by differentially sponging miR-200b/c and let-7b. Sci Signal 10.

Zhu, L., Yang, N., Du, G., Li, C., Liu, G., Liu, S., et al. (2018). LncRNA CRNDE promotes the epithelial-mesenchymal transition of hepatocellular carcinoma cells via enhancing the Wnt/β-catenin signaling pathway. J Cell Biochem 120 1156-1164.
